# Cellular RNA and DNA sensing pathways are essential for the dose-dependent response of human monocytes to ionizing radiation

**DOI:** 10.3389/fimmu.2023.1235936

**Published:** 2023-12-12

**Authors:** Natallia Mikhalkevich, Eric Russ, Sergey Iordanskiy

**Affiliations:** ^1^ Department of Pharmacology & Molecular Therapeutics, Uniformed Services University of the Health Sciences, Bethesda, MD, United States; ^2^ The Henry M. Jackson Foundation for the Advancement of Military Medicine, Bethesda, MD, United States; ^3^ The American Genome Center (TAGC), Collaborative Health Initiative Research Program, Uniformed Services University of the Health Sciences, Bethesda, MD, United States; ^4^ Graduate Program of Cellular and Molecular Biology, Uniformed Services University of the Health Sciences, Bethesda, MD, United States; ^5^ Armed Forces Radiobiology Research Institute, Uniformed Services University of The Health Sciences, Bethesda, MD, United States

**Keywords:** monocytes, macrophages, gamma radiation, RIG-I-like receptors (RLRs), melanoma differentiation-associated protein 5 (MDA-5), stimulator of interferon genes (STING), MAVS, RNA:DNA hybrids

## Abstract

Circulating monocytes are important players of the inflammatory response to ionizing radiation (IR). These IR-resistant immune cells migrate to radiation-damaged tissues and differentiate into macrophages that phagocytize dying cells, but also facilitate inflammation. Besides the effect of damage-associated molecular patterns, released from irradiated tissues, the inflammatory activation of monocytes and macrophages is largely dependent on IR-induced DNA damage and aberrant transcriptional activity, which may facilitate expression of type I interferons (IFN-I) and numerous inflammation-related genes. We analyzed the accumulation of dsRNA, dsDNA fragments, and RNA:DNA hybrids in the context of induction of RNA-triggered MAVS-mediated and DNA-triggered STING-mediated signaling pathways, in primary human monocytes and a monocytic cell line, THP1, in response to various doses of gamma IR. We found that exposure to lower doses (<7.5 Gy) led to the accumulation of dsRNA, along with dsDNA and RNA:DNA hybrids and activated both MAVS and STING pathway-induced gene expression and signaling activity of IFN-I. Higher doses of IR resulted in the reduced dsRNA level, degradation of RNA-sensing mediators involved in MAVS signaling and coincided with an increased accumulation of dsDNA and RNA:DNA hybrids that correlated with elevated STING signaling and NF-κB-dependent gene expression. While both pathways activate IFN-I expression, using MAVS- and STING-knockout THP1 cells, we identified differences in the spectra of interferon-stimulated genes (ISGs) that are associated with each specific signaling pathway and outlined a large group of STING signaling-associated genes. Using the RNAi technique, we found that increasing the dose of IR activates STING signaling through the DNA sensor cGAS, along with suppression of the DDX41 helicase, which is known to reduce the accumulation of RNA:DNA hybrids and thereby limit cGAS/STING signaling activity. Together, these results indicate that depending on the applied dose, IR leads to the activation of either dsRNA-induced MAVS signaling, which predominantly leads to the expression of both pro- and anti-inflammatory markers, or dsDNA-induced STING signaling that contributes to pro-inflammatory activation of the cells. While RNA:DNA hybrids boost both MAVS- and STING-mediated signaling pathways, these structures being accumulated upon high IR doses promote type I interferon expression and appear to be potent enhancers of radiation dose-dependent pro-inflammatory activation of monocytes.

## Introduction

Exposure to ionizing radiation (IR) that occurs in expected (i.e., radiation therapy or space travel) and unexpected (i.e., nuclear weapons detonation, or nuclear power plant accidents) scenarios presents a formidable clinical challenge. Whole-body or significant partial-body exposures to IR in many cases are associated with inflammation due to an acute upregulation of inflammatory mediators and sustained hyperactivity of the cells that produce them (1, 2). If not resolved, irradiated tissues develop chronic inflammation and fibrosis, considered the most severe side effects of radiation therapy (3–6).

Circulating monocytes have a relatively short lifespan, from 1 to 7 days, depending on the subset (7). Classical (CD14^++^, CD16^−^) monocytes represent the majority (85–90%) of the whole monocyte population, consisting of cells that express CD14, a co-receptor with Toll-like receptor 4 (TLR4) and lymphocyte antigen 96 (MD2) that together detect LPS ligand, but not CD16. These cells are associated with inflammatory responses and circulate in the blood for only one day until their extravasation into the tissues and differentiation into pro-inflammatory macrophages (7–9). Whereas other subsets of the monocyte population, the intermediate (CD14^++^, CD16^+^) and nonclassical (CD14^+/low^, CD16^++^) monocytes, circulate for 4 and 7 days, respectively and are associated with the activation of anti-inflammatory/profibrotic pathways (9).

Monocytes are intrinsically more radioresistant than other circulating leukocytes and even after exposure to 20-40 Gy doses of γIR retain ~60% viability for up to a week (10, 11). Multiple *in vitro* and *in vivo* studies indicate that the damage-associated molecular patterns (DAMPs) and cytokines released from IR-damaged tissues, as well as radiation itself activate and recruit circulating monocytes to damaged areas and promote their differentiation into macrophages, a highly radioresistant cell population (12–16). While macrophages are essential for normal immune function, they are also believed to play a significant role in the early overactive pro-inflammatory response and in the delayed anti-inflammatory/pro-fibrotic changes that occur in irradiated tissues (17–19). To fulfill these diverse activities, macrophages are variably “polarized,” leading to several potential phenotypes, including the traditional pro- and anti-inflammatory phenotypes (M1 and M2, respectively), as well as more complex phenotypes with mixed features (12, 20).

Activation of monocytes to the differentiation and polarization towards an M1 macrophage phenotype by IR is believed to be a driving factor in acute radiation-induced inflammation, developing early after radiation exposure. Besides DAMPs, which are released by radiation-damaged cells and activate monocytes via surface receptor signaling (5, 17, 21), IR itself also contributes to their phenotypic changes via the production of ROS and nitric oxide (22, 23) and radiation-induced DNA strand breaks, which lead to the accumulation of DNA fragments, detected by cyclic GMP-AMP (cGAMP) synthase (cGAS) and its downstream adaptor, stimulator of interferon genes (STING) (24). Together, these mechanisms stimulate pro-inflammatory signaling (25–27). However, we and others (12, 28) have shown that in monocytes and monocyte-derived macrophages (MDM), IR induces mixed polarity because, along with the activation of NF-κB-dependent cytokine expression, it also promotes IRF3-induced expression of type I interferons (IFN-I), IFNα and IFNβ and, hence up-regulating interferon-stimulated genes (ISGs), resulting in the expression of pro-inflammatory along with some anti-inflammatory markers. IR doses from 0.5 to 5 Gy that do not cause massive DNA damage promote the transcription of human endogenous retroviruses (HERVs), an abundant source of transposable elements comprising more than 8% of the human genome, in the sense and antisense directions, resulting in cytoplasmic accumulation of viral dsRNA. This RNA binds to the dsRNA sensors MDA5 and TLR3 and induces signaling pathways that trigger IFN-I and the expression of ISGs, including both pro- and anti-inflammatory modulators (28). This suggests an important role of both RNA- and DNA-sensor-induced pathways in the innate immune response of monocytes and MDMs to IR. However, which signaling mechanisms play a critical role in the dose-dependent activation and inflammatory response of monocytes and MDMs to IR, specifically the initial differentiation of irradiated monocytes into particular pro-/anti-inflammatory phenotype, remains unclear.

In the present study, we investigate the nucleic acid-induced signaling pathways that are involved in changes of the gene expression profile of human monocytes and macrophages in the context of their dose-dependent response to gamma radiation. Using primary human monocytes and the monocytic cell line, THP1, we demonstrate that raising the γIR dose drives a change in the phenotypic profiles of these cells, determined by a decreasing participation of RNA-induced (RIG-I, MDA5/MAVS) signaling along with an increasing role of the DNA-induced (cGAS/STING) signaling pathway, which is enhanced by RNA:DNA heteroduplex-triggered signaling, that together result in differences in the spectra of interferon-stimulated genes and inflammatory mediators.

## Results

### Human monocytes are radio-resistant and are activated in response to increasing doses of gamma radiation

**Figure 1:**
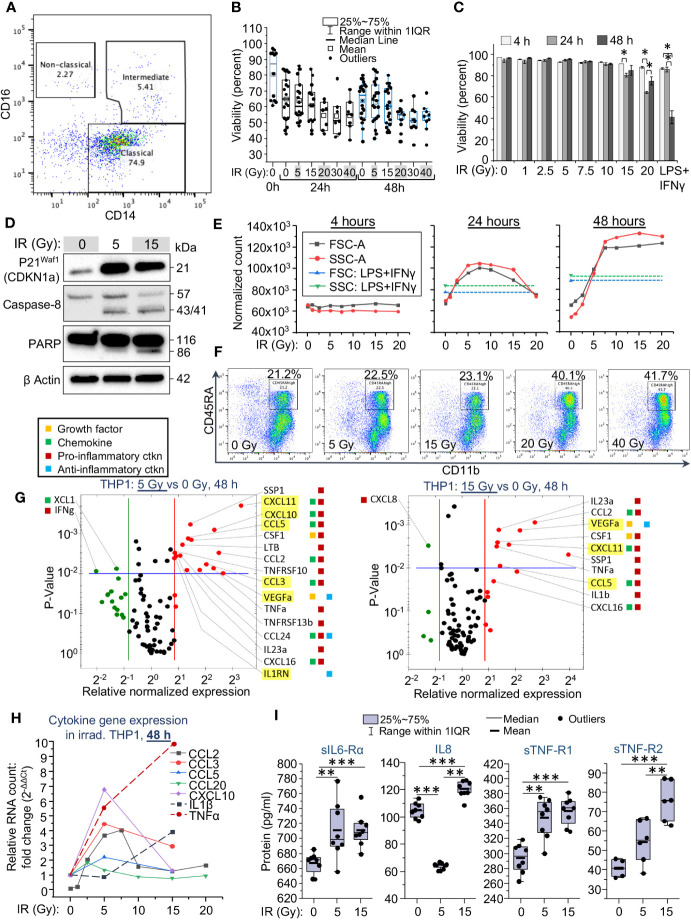
Cytomorphological and functional characteristics of monocytes are changing depending on the applied dose of gamma radiation. **(A)** Analysis of monocyte population in fraction 5 of elutriated human PBMCs profiled by flow cytometry, shown for 1 representative of 5 tested donors. Lineage markers were used to exclude contamination with lymphocyte subsets. Lineage negative (CD3-, CD19-, CD20-, CD56-, CD66b-) and viable (DAPI-negative) single cells were labeled with anti-CD14-APC and anti-CD16-PE coupled antibodies to outline subpopulations of classical, intermediate and non-classical monocytes. **(B)** Viability of primary human monocytes measured by flow cytometry as a percentage of DAPI-negative single cells in the total population, 48 h after exposure to indicated doses of γIR. Error bars indicate ± SD of monocyte populations from five different donors; 4 replicates of each – paired Wilcoxon test. **(C)** Viability of THP1 cells measured by flow cytometry as a percentage of DAPI-negative cells in the total population, 4, 24, and 48 h after exposure to indicated doses of γIR. Error bars indicate ± SD of three independent biological replicates; * *p*<0.01 – paired *t* test. **(D)** Western blot analysis of senescence marker p21^Waf1^ (CDKN1A) protein and apoptosis markers, cleaved caspase-8 and PARP proteins in THP1 lysates, 48 h after irradiation with 5 or 15 Gy doses. Forty μg of total protein were loaded. The β actin loading control of the panels is the same, since proteins were separated in the same gel electrophoresis. **(E)** Change of the median values of cell size (FSC-A) and granularity (SSC-A) of THP1 cell populations depending on exposure to indicated γIR doses, measured 4, 24, and 48 h post-exposure; control – LPS (100 ng/mL) + IFNγ (25n/mL) -treated cells. Error bars indicate ± SD of 3 independent biological replicates. **(F)** Analysis of the surface expression of myeloid cell marker CD45RA vs. macrophage marker CD11b in peripheral blood monocytes, exposed to indicated doses of γIR, 48 h post-exposure. Lineage markers were used to exclude contamination with lymphocyte subsets. Lineage negative (CD3^-^, CD19^-^, CD20^-^, CD56^-^, CD66b^-^) and viable (DAPI-negative) single cells were labeled with anti-CD45RA-APC and anti-CD11b-PE coupled antibodies. **(G)** Scatter plots showing cytokine expression in 5 Gy (top) and 15 Gy (bottom) irradiated vs. control THP1 cells, measured by PCR array of total cellular RNA samples, 48h post-IR. Red dots indicate twofold increase in gene expression in irradiated cells vs non-irradiated cells, green dots indicate twofold decrease in expression. The area above horizontal blue line indicates p < 0.01. SASP marker genes are highlighted by yellow background. The colored squares on the right mean: yellow – activity as a growth factor, green – chemokine, red – pro-inflammatory, blue – anti-inflammatory activities. **(H)** Quantitation of SASP chemokines (solid lines) and pro-inflammatory cytokines (dash lines) mRNA in THP1 cells exposed to indicated doses of γIR using RT-qPCR, 48h after γIR. Error bars indicate ± SD of three independent biological replicates. **(I)** Quantification of soluble inflammation markers in culture media of THP1 cells exposed to the indicated γIR doses using Bio-Plex multiplex immunoassay, 48h after γIR. Minimum number of biological samples is 3. * *p*<0.05, ** *p*<0.01, *** *p*<0.001, paired Wilcoxon test.

While monocytes are known to be radiation-resistant immune cells (14, 29), we first had to evaluate the specific effect of increasing doses of γIR on the phenotype of human monocytes. We analyzed primary human monocytes, isolated from the peripheral blood mononuclear cells (PBMCs) of healthy donors at the NIH Blood Bank, using the Elutra system (30), and the human monocytic cell line, THP1. The population of cells collected from fraction 5 after elutriation express major monocytic surface markers (**Supplementary Figure 1A**) that are consistent with published data, indicating that the median proportion of monocytes in this fraction is 88% (31). Our further analysis showed that the proportion of classical (CD14^++^, CD16^−^), intermediate (CD14^++^, CD16^+^), and non-classical (CD14^+/low^, CD16^++^) monocytes is also consistent with the typical phenotypic distribution within circulating monocytes (32) (**Figure 1A**).

In our experiment, primary monocytes showed resistance to γIR. The median viability of the cells decreased from 85 to 65% during the first 24 h in culture without irradiation due to a short lifespan of classical monocytes (7–9) (**Supplementary Figure 1B**, left panel). However, exposure to doses from 1 to 40 Gy has not changed significantly the viability of primary monocytes depending on the dose within 48 h. Even after the maximal dose, the cell viability did not fall below 50% (**Figure 1B**). The viability of monocytes seemed more dependent on the particular donor than on the dose of IR. According to Patel and coauthors (7), classical monocytes, after release from the bone, circulate for a day, and then either die or extravasate into the surrounding tissues, while some of them sequentially differentiate into intermediate and nonclassical cells. Our data indicate that exposure to γIR additionally contributes to the depletion of the classical monocyte subpopulation, but at the same time leads to a remarkable increase in the proportion of intermediate ones (**Supplementary Figure 1B**).

Due to the high heterogeneity of primary monocytes, pointed out in multiple studies (33–35), we examined the sensitivity to radiation of a well-studied human monocytic cell line, THP1. Within 48 h after IR, these cells did not show remarkably reduced viability when exposed to doses from 1 to 10 Gy (**Figure 1C**). The doses of 15 and 20 Gy, as in primary monocytes, resulted in a 15-25% reduction in the number of viable THP1 cells. By contrast to primary monocytes, doses higher than this resulted in significant death within 48 h, likely due to the actively dividing nature of THP1 cells compared to primary monocytes. Interestingly, incubation with LPS and IFNγ, well-known inducers of a pro-inflammatory phenotype (36, 37), also reduced the viability of THP1 cells by 50%. Of note, in irradiated cells, such a decrease in viability is observed eight days after γIR with a dose of 15 Gy (**Supplementary Figure 1C**). Based on these results, we further assessed the phenotypic characteristics of THP1 cells in response to γIR doses of 1-20 Gy. While monocytes continue to be viable after IR exposure for a long time, the effects of radiation related to DNA damage and oxidative stress cause cell cycle arrest and induce senescence. Analysis of CDKN1A (p21^Waf1^) expression, a hallmark of senescence-associated cell cycle arrest (38, 39), revealed a remarkable increase in the level of this protein in THP1 cells 48 h after γIR exposure (**Figure 1D**). Consistently, the level of cleavage of caspase 8 and PARP proteins, which indicate the progression of apoptosis, was detected in 5 Gy-irradiated cells, but was highest in the cells exposed to a 15 Gy dose.

Circulating monocytes are shown rapidly changing their size and granulation in response to the pathogen-associated molecular patterns (PAMPs), DAMPs and cytokines, that induce their pro-inflammatory activation and differentiation into monocyte-derived macrophages (9, 17–19, 40). To assess the correlation of these parameters with the applied γIR doses, we tested the size and granularity of THP1 cells and primary monocytes. No changes were detected at 4 h after IR (**Supplementary Figures 2A, C**, **Figure 1E**). Exposure of THP1 cells to doses from 1 to 7.5 Gy led to remarkable, sequential increases in both parameters measured 24 h and especially 48 h post-IR (**Supplementary Figures 2A–C**, **Figure 1E**). After 48 hours, cells exposed to higher doses (10 to 20 Gy) matched these cytomorphological characteristics of the cells exposed to 7.5 Gy. The median size and granulation of these cells exceeded those of cells treated with LPS and IFNγ.

Primary monocytes, which are quite resistant to even the maximal tested IR doses, being exposed to increasing doses of γIR, also derived a subset of cells with increased size and granularity. The proportion of these cells raised from 2.5% in the unexposed population to 12.8% in the population of 20 Gy irradiated monocytes (**Supplementary Figure 3A**). Analysis of the surface expression of a myeloid cell marker CD45RA, regarded as an activation marker of peripheral blood monocytes (41, 42), shows a restrained increase in response to doses of 5 and 15 Gy, and a remarkable twofold elevation after exposure to 20 and 40 Gy (**Figure 1F**). A similar trend is observed in the expression of an activation marker, HLA-DR and the inflammatory macrophage marker, CD86 (**Supplementary Figures 3B, C**), indicating that primary monocytes are activated by γIR to differentiate towards pro-inflammatory macrophages after exposure to doses of 20 Gy and above.

To test whether radiation-related cytomorphological changes of monocytes correlate with their cytokine profile, we measured cytokine expression in THP1 monocytes exposed to 5 and 15 Gy doses of γIR at 48 h and 72 h time points. Quantification of mRNA indicated that the expression of many cytokines was significantly more than doubled after a 5 Gy dose at 48 h post-IR (**Supplementary Table 1**). Many overexpressed chemokines and interleukins are regarded as markers of senescence-associated secretory phenotype (SASP) (43, 44), highlighted in yellow in **Figure 1G**. The SASP-related chemokines CCL3 (MIP-1α), CCL5 (RANTES), CXCL10 (Mob-1), and CXCL11 (I-TAC) were detected to be upregulated, as well as IL-1β, TNFα, and IL23, the hallmarks of a robust inflammatory response. While most of the SASP-released factors exhibit pro-inflammatory activity (45), they are also known to enhance resistance to apoptosis, reduce proliferative potential, and alter the immune response of both irradiated and unexposed cells (46, 47). Indeed, among the sixteen cytokines whose activity is remarkably increased after a 5 Gy dose of γIR, and 10 cytokines upregulated after 15 Gy, 90% are known to be pro-inflammatory factors.

At the 72-h time point, a dramatic increase in the expression of distinct pro-inflammatory cytokines, such as IL6 (22x-28x increase), IL22 (6x-8x), and CCL8 (4.5x-9.5x) was detected. Interestingly, multiple anti-inflammatory modulators (IL10, IL13, MSTN, TGFβ) were also found to be upregulated. Along with this, a portion of pro-inflammatory cytokines (CCL20, IL18, CXCL10) was downregulated (**Supplementary Figure 4A**, **Supplementary Table 1**). The expression of certain SASP-associated chemokine markers was increased (CCL8, CCL13, CCL11, IL13, CCL1), whereas some of them, on the contrary, were downregulated (CCL20, CXCL10). This suggests that, at a later time point, irradiated monocytes tend to change towards a complex phenotype with an elevated expression of both pro-inflammatory and anti-inflammatory, pro-fibrotic markers.

To analyze the correlation between the expression of SASP markers and robust pro-inflammatory cytokines in response to gradually increased doses of γIR, we measured the expression of signature genes after THP1 cells were exposed to doses of γIR used in the viability experiments. Of note, the SASP-associated chemokines CCL2 (MCP-1), CCL3 (MIP-1α), CCL5 (RANTES), and CXCL10 (IP-10) were found upregulated 48 h after doses of 5-7.5 Gy, whereas the highest level of expression of IL-1β and TNFα was detected after 15 Gy dose of IR (**Figure 1H**). Further analysis of the secretion of inflammation-related factors by irradiated THP1 cells, performed using a multiplex immunoassay (**Figure 1I**), revealed a significantly increased level of soluble IL6 receptor subunit α, an agonist of IL6 activity (48), and TNF receptors 1 and 2. Concentration of the last one is increased in accordance with the IR dose. The characteristic monocyte/macrophage produced chemokine IL8 (CXCL8), one of the major mediators of the inflammatory response, was found upregulated in response to 15 Gy dose, whereas lower IR dose reduced its level in the culture media.

Among the tested cytokines in primary monocytes, only the chemokine CCL2 (monocyte chemoattractant protein-1, MCP-1) demonstrated a significant and stable increase in expression within the first 24 h after IR at doses from 5 to 40 Gy (**Supplementary Figure 4B**). After 48 h, the expression of this chemokine was significantly upregulated in the cells exposed to 5 and 15 Gy, but not higher doses of γIR, similarly to the results from THP1 cells. Since CCL2 is one of the key chemokines that activate monocytes/macrophages to perform migration and tissue infiltration and its expression is associated with various inflammatory diseases (49), upregulation of this gene in irradiated monocytes may be an additional piece of evidence that γIR results in the induction of differentiation of these cells towards pro-inflammatory macrophages.

### Gamma radiation activates intracellular RNA and DNA sensors in a dose-dependent manner

**Figure 2:**
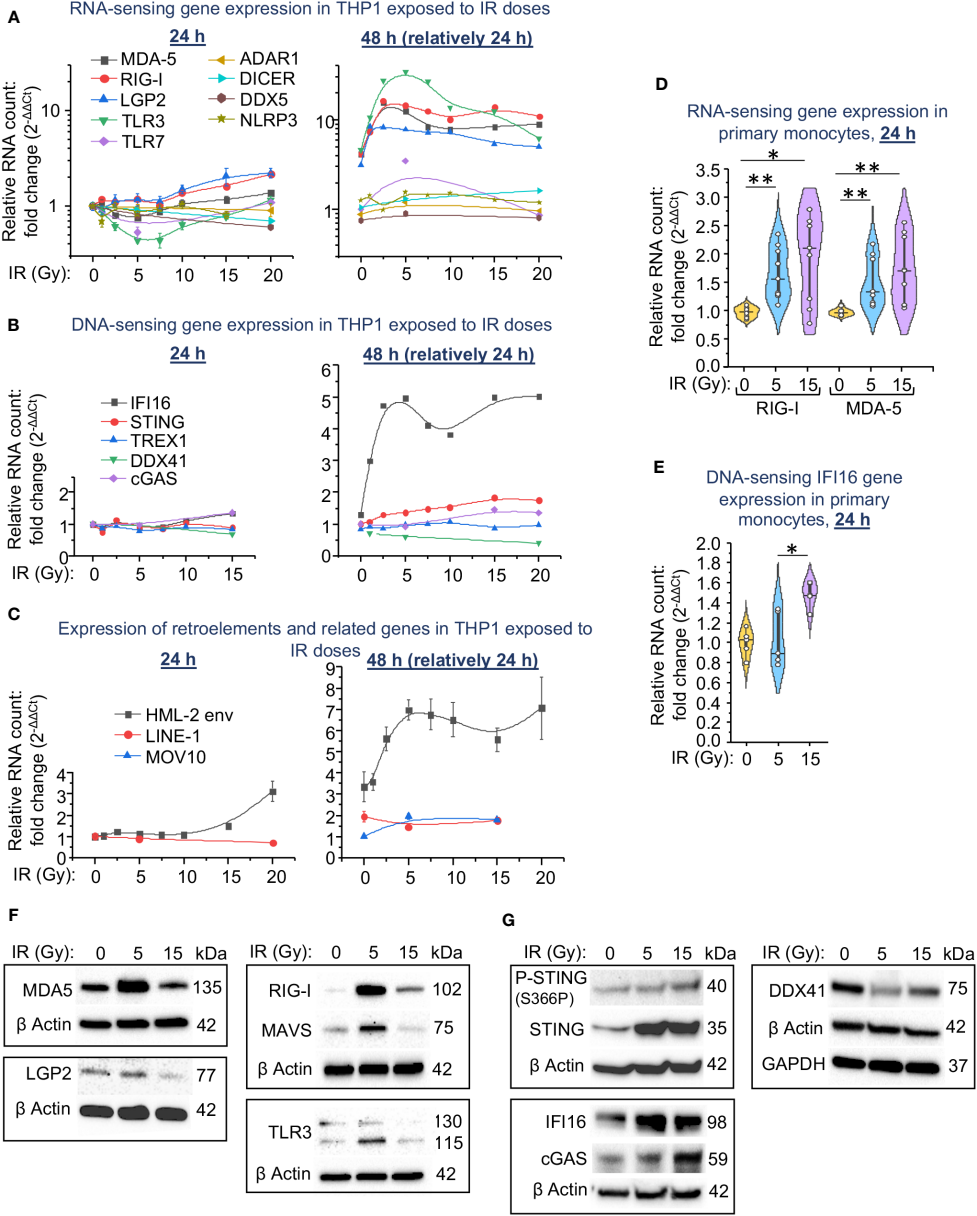
Expression of intracellular RNA and DNA sensors and their signaling pathway components in THP1 cells and primary monocytes depends on the irradiation dose. **(A-C)** Expression of genes encoding RNA sensors and important RNA-binding proteins involved in activation of IFN-I response **(A)**, encoding STING pathway components and TREX1 DNA exonuclease **(B)**, and HERK HML-2 *env* gene, LINE1 retrotransposons and MOV10 RNA helicase **(C)** in THP1 cells exposed to indicated doses of γIR, measured by RT-qPCR vs. GAPDH and GUSB reference housekeeping genes at 24 and 48 h time-points. Error bars: ± SD of three independent biological replicates. **(D, E)** Expression of genes encoding RNA sensors RIG-I and MDA-5 **(D)** and STING pathway component IFI16 **(E)** in primary human monocytes exposed to indicated doses of γIR, measured by RT-qPCR vs. GAPDH and GUSB reference housekeeping genes at 24 h time-point. Violin plots of RT-qPCR measured expression of n = 6 is shown. * *p*<0.05, ** *p*<0.01, paired Wilcoxon test. **(F, G)** Immunoblotting of RNA sensors MDA-5, RIG-I, LGP2, RLR adaptor MAVS, and TLR3 **(F)** and STING pathway components IFI16, cGAS, DDX41, STING and phospho-STING (S366P) **(G)** in THP1 cells, 48h after exposure to 5 and 15 Gy of γIR (40 μg of total protein).

Many inflammation-related factors that are upregulated in monocytes upon IR are encoded by a group of interferon-stimulated genes that are activated via the JAK/STAT pathway upon induction by interferons (50, 51). To identify the signaling mechanisms that underly monocyte activation and differentiation, we analyzed the expression of cytoplasmic sensors of RNA and DNA molecules, which are major triggers of an interferon response, and the adaptors of downstream pathways that mediate the expression of interferons and numerous genes activated by the transcription factor NF-κB. Within the first 24 h, no remarkable changes in the expression of RNA receptors (TLR3, TLR7, RIG-I, MDA5, and LGP2) or DNA sensors (cGAS, IFI16, DDX41) and their adaptor STING, were detected in THP1 cells exposed to IR doses less than 10 Gy (**Figures 2A, B**, left panels). The doses from 10 to 20 Gy led to a moderately (2-2.5-fold) increased expression of the RNA receptor genes RIG-I and LGP2, members of the RIG-I-like receptor (RLR) family. However, after 48 hours, we observed a trend towards a sharp increase in the expression of both DNA and RNA sensors in response to doses of γIR of 1 Gy and higher. The DNA sensor IFI16, as well as all RLRs and the dsRNA receptor TLR3, were dramatically upregulated, whereas the expression of TLR7, a ssRNA sensor, was only threefold increased after a 5 Gy dose (**Figures 2A, B**, right panels). Notably, the expression of genes encoding ADAR1 and DICER, proteins responsible for protecting cells from dsRNA accumulation (52), was not found altered. This correlated with transcriptional elevation of endogenous retrovirus HERVK HML-2 (**Figure 2C**), which was previously found among the group of HERVs that are upregulated upon IR and is able to activate both MDA5 and TLR3 signaling (28). At the same time, another large group of retroelements, LINE-I, was not found upregulated after the tested doses of IR. Interestingly, expression of the genes encoding the RNA helicase MOV10 that protects cells from LINE1 retrotransposition (53–55), and TREX1 DNA exonuclease, which is responsible for the processing of DNA fragments from endogenous retroelements, including LINE1, SINE elements and HERVs (56), were not found upregulated (**Figures 2B, C**). This might also contribute to the activation of RNA- and DNA-sensing pathways by the products of HERV expression.

Transcription of the RNA receptors RIG-I and MDA-5 was also significantly upregulated in primary human monocytes in the first 24 h after exposure to IR. The median expression level in response to a dose of 15 Gy was slightly higher than in response to a 5 Gy dose of γIR (**Figure 2D**). Expression of the DNA sensor IFI16 was significantly upregulated by 1.5-fold only after exposure of monocytes to a 15 Gy dose (**Figure 2E**).

Analysis of cytoplasmic RNA and DNA sensors on a protein level revealed a remarkable increase of the major dsRNA receptor MDA5 in THP1 cells exposed to a 5 Gy γIR, whereas exposure to a 15 Gy dose reduced the quantity of this protein (**Figure 2F**). A receptor of short dsRNA fragments and 5′-triphosphorylated ssRNA, RIG-I (57), demonstrates the same trend. Another cytosolic RNA sensor LGP2, the N terminally-truncated RLR involved in regulation of MDA5 and RIG-I signaling (58, 59), is also found reduced in the 15 Gy-exposed cells. Finally, the endosomal dsRNA receptor TLR3 was increased upon 5 Gy IR, but decreased after the 15 Gy dose as well. Interestingly, we did not observe this effect at the mRNA level, where the expression of RNA-sensing molecules was maximally increased at 5 Gy IR and then did not change significantly between 5 and 20 Gy. This may be determined by either the regulation of the expression of these genes at the post-transcriptional level or by the instability of mitochondrial membrane-bound components of the MAVS pathway at high doses of radiation.

Analysis of the components that constitute the DNA-sensing signaling pathway shows that the cytosolic adaptor of DNA-sensing signaling, STING, was remarkably increased in all irradiated cells. However, Ser366 phosphorylation, a hallmark of STING activation (60), was increased proportionally the IR dose and reached the highest level in 15 Gy-irradiated cells (**Figure 2G**). The elevated level of the major dsDNA sensor cGAS that induces STING signaling was also found upregulated 48 h after IR, especially after the higher dose of 15 Gy. The similar trend was observed for the DNA sensor IFI16, a non-canonical activator of the STING pathway that has also been shown to induce NF-κB-dependent gene expression in response to DNA damage (61). While the maximum level of this protein was detected after the medium IR dose (5 Gy). Finally, presentation of another nucleic acid-binding molecule, the DEAD/H box helicase DDX41, which has been found involved in STING signaling activation upon different DNA virus infections (62, 63), was decreased upon IR, especially after 5 Gy dose.

Together, our data indicate a correlation in the expression of the dsRNA sensors and the adaptor protein MAVS during the response of monocytes to medium IR doses (beneath 7.5 Gy for THP1 cells). The higher doses correlate with an increased level of the markers of DNA damage-related signaling pathway, mediated by the adaptor protein STING. Notably, this response is delayed and can be detected on the second day after IR, suggesting an accumulation of either dsRNA or the products of DNA damage to activate the respective signaling pathways.

### Radiation changes the ratio of nucleic acid substrates in monocytes in a dose-dependent manner

**Figure 3:**
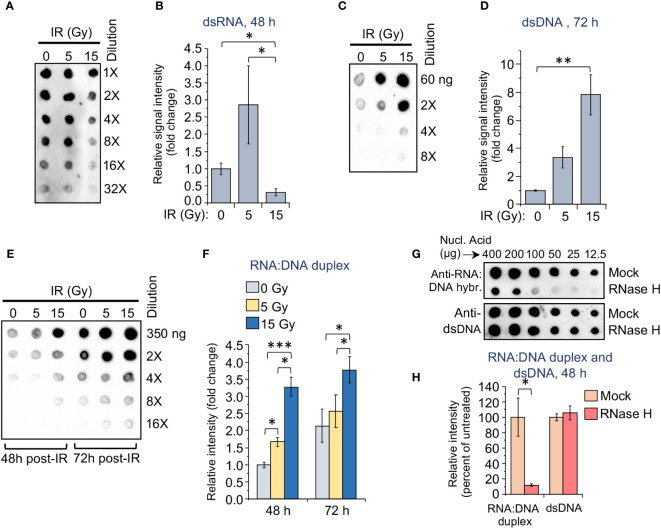
The accumulation of dsRNA, dsDNA, and RNA:DNA heteroduplexes in monocytes differs depending on the applied dose of gamma radiation. **(A)** Dot-blot loaded with RNA extracts from the radiation-exposed (5 or 15 Gy) and control THP1 cells. RNA samples were loaded onto nylon membranes in a dilution series of 200, 100, 50, 25, 12.5, and 6.25 ng per 2 μl dot. Membranes were then probed with 9D5 antibody, recognizing dsRNA (Absolute Antibody). **(B)** Grayscale values of the dots in the dot blot shown in **(A)**, quantified using ImageJ software, and normalized to initial nucleic acid concentration in each sample. Results are presented as a ratio of analyzed samples to the sample from unexposed cells. Error bars indicate ± SD of six independent measurements; * *p*<0.05, – paired *t* test. **(C)** Dot-blot loaded with DNA extracts from the radiation-exposed (5 or 15 Gy) and control THP1 cells. DNA samples were loaded onto nylon membranes in a dilution series of 60, 30, 15, and 7.5 ng per 2 μl dot. Membranes were then probed with anti-dsDNA antibody, recognizing dsDNA fragments. **(D)** Grayscale values of DNA dots in the dot blot shown in **(C)**, quantified as described in **(B)**. Error bars indicate ± SD of four independent measurements: ** *p*<0.01, – paired *t* test. **(E)** Dot-blot loaded with nucleic acid extracts from the radiation-exposed (5 or 15 Gy) and control THP1 cells, 48 and 72 h post-IR. DNA samples were loaded in a dilution series of 350, 175, 87.5, 43.75, and 22 ng per 2 μl dot. Membranes were then probed with S9.6 antibody, recognizing RNA:DNA heteroduplexes (Kerafast). **(F)** Grayscale values of RNA:DNA dots in the dot blot shown in **(E)**, quantified as described in **(B)**. Error bars indicate ± SD of five independent measurements: *** *p*<0.001, ** *p*<0.01, * *p*<0.05, – paired *t* test. **(G)** Dot-blot loaded with nucleic acid extracts from the 5 Gy radiation-exposed THP1 cells, 72 h post-IR. The samples were either mock treated or treated with RNase H, then loaded onto nylon membranes in indicated dilution series. Membranes were then probed with either S9.6 antibody (upper panel) or anti-dsDNA antibody (lower panel). **(H)** Grayscale values of RNA:DNA and dsDNA dots in the dot blot panels shown in **(G)**, quantified as described in **(B)**. Error bars indicate ± SD of six independent measurements: * *p*<0.05, – paired *t* test.

To identify a potential molecular mechanism underlying the differential upregulation of dsRNA or DNA sensors in response to various doses of IR in monocytes, we examined the accumulation of dsRNA, dsDNA and RNA:DNA heteroduplexes in THP1 cells exposed to different γIR doses. While evaluation of dsRNA in total cell lysates using the 9D5 antibody displayed high variability among the samples, we could detect a trend towards an increased accumulation of dsRNA in the cells irradiated with a 5 Gy dose, and a significant reduction of the signal in the 15 Gy-exposed cells (**Figures 3A, B**). Since dsDNA molecules in the cytoplasm of monocytes can activate the cGAS/STING signaling pathway upon high IR doses, we assessed fragments of dsDNA in irradiated THP1 using specific antibody. Predictably, the doses of 5 and especially 15 Gy led to respectively three- and sevenfold increases in the level of dsDNA fragments, as compared with unexposed cells (**Figures 3C, D**). Based on earlier publications, indicating that RNA:DNA hybrids can also activate the cGAS/STING pathway (64–66), we measured accumulation of these heteroduplexes in the irradiated cells. Analysis with S9.6, an antibody that recognizes RNA:DNA heteroduplexes, revealed a 1.7-fold increase in the abundance of these hybrid molecules in the cells exposed to 5 Gy IR, and a threefold increase in 15 Gy-exposed cells within 48 h after irradiation. A similar trend continues over 72 hours (**Figures 3E, F**). Treatment of the serial dilutions of nucleic acid samples from 5 Gy-irradiated cells with RNase-H, which destroys the RNA strand in RNA:DNA duplexes, led to a dramatic reduction of the signal in the immunoblot with the S9.6 antibody (**Figures 3G, H**). The anti-dsDNA antibody control did not reveal differences between RNase H treated and untreated samples. Given that the S9.6 antibody from Kerafast used for this analysis also cross-reacts with dsRNA (~5-fold less efficient than with heteroduplexes), a dramatic decrease of the signal after RNase H treatment suggests that the majority of nucleic acid structures detected with this antibody represent RNA:DNA hybrids.

### MAVS- and STING-mediated pathways differently affect expression of type I interferons, NF-kB-activated and interferon-stimulated genes depending on the radiation dose

**Figure 4:**
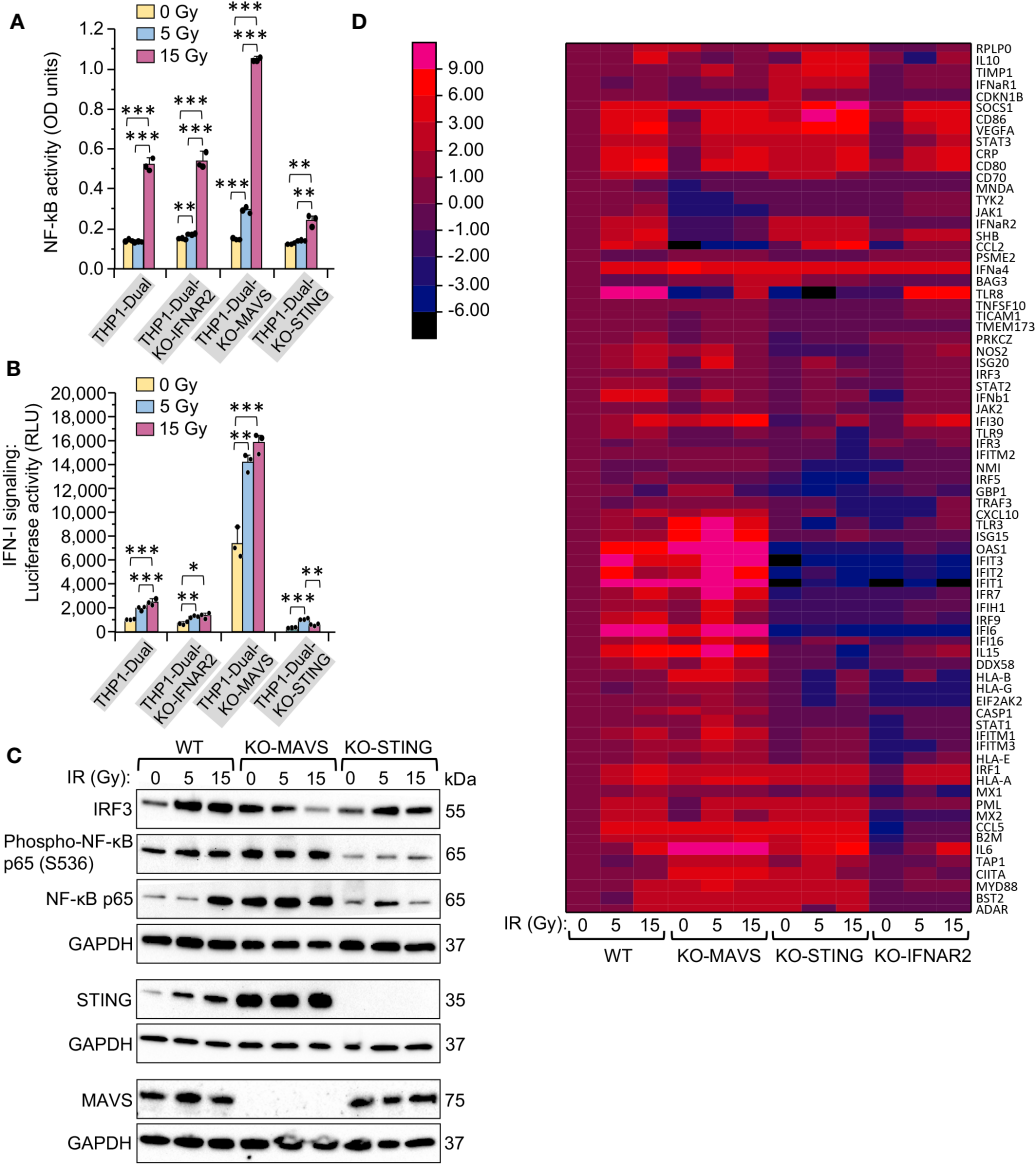
MAVS- and STING-mediated signaling pathways are activated differently in response to the same doses of gamma radiation in monocytes. **(A, B)** Activation of NF-κB-dependent transcription **(A)** and type I interferon-induced IFNAR signaling **(B)** in unexposed and exposed to 5- or 15-Gy γIR doses reporter THP1-Dual, THP1-Dual KO-IFNAR2, THP1-Dual KO-MAVS and THP1-Dual KO-STING cells. Parental THP1-Dual cells and derived knockout cell lines express SEAP gene driven by an IFN-β minimal promoter activated by NF-κB and secrete Lucia luciferase gene under the control of an IFNAR signaling-activated ISG54 minimal promoter; THP1-Dual KO-IFNAR2, THP1-Dual KO-MAVS and THP1-Dual KO-STING cells are generated from THP1-Dual cells by stable knockout of the IFNAR2 receptor, MAVS and STING nucleic acid pathway adaptors respectively. After 48 h of incubation and staining of culture media with either SEAP-sensitive QUANTI-Blue dye with subsequent measurement of the absorbance (600 nm) **(A)** or staining with QUANTI-Luc substrate and measurement of luminescence **(B)** were performed for each sample. Data were normalized to non-irradiated controls. Error bars indicate ± SD of a minimum of 3 independent biological replicates; *** *p ≤* 0.001, ** *p ≤* 0.01, * *p ≤* 0.05; paired *t* test. **(C)** Immunoblotting of transcription factors IRF3, NF-κB p65 and Phospho-S536 NF-κB p65 subunit, and STING and MAVS in lysates of THP1-Dual, THP1-Dual KO-MAVS and THP1-Dual KO-STING cells exposed to indicated γIR doses, 48 h post-exposure; 20 μg of total protein were loaded. The GAPDH loading control of the panels comprised by IRF3, Phospho-NF-κB p65 and NF-κB p65 is the same, since proteins were separated in the same gel electrophoresis. **(D)** Heatmap depicting expression of interferon-stimulated genes (ISGs) in THP1-Dual, THP1-Dual KO-MAVS, THP1-Dual KO-STING, and THP1-Dual KO-IFNAR2 cells exposed to a 5 or 15 Gy γIR dose and non-irradiated cells, measured by RT^2^ PCR array of total cellular RNA samples, 48h after γIR exposure: rows: ISG codes; columns: samples. Color codes are shown on the separate left panel.

To assess the involvement of RNA-induced MAVS-mediated signaling and DNA-induced STING-mediated pathway in the activation of IRF3-dependent expression of IFN-I and thereby ISGs, as well as in the expression of NF-κB-dependent genes in response to an increase in γIR doses, we tested autocrine IFNα and IFNβ signaling and NF-κB-dependent reporter gene expression in THP1-Dual reporter cells. This engineered cell line expresses secreted an embryonic alkaline phosphatase gene driven by a recombinant promoter activated by NF-κB and the Lucia luciferase gene activated by signaling from interferon-α/β receptor (IFNAR). Another reporter cell line, THP1-Dual KO-IFNAR2, generated from the THP1-Dual cells by a stable knockout of the IFNAR subunit 2, was used as a negative control to test the IFN-I stimulated response. The cell lines THP1-Dual KO-MAVS and THP1-Dual KO-STING, which are also derived from the parental THP1-Dual cells and have impaired expression of these genes, were analyzed to the evaluate contribution of RLRs, which transduce signaling through the adaptor protein MAVS, and to evaluate the contribution of STING-mediated signaling, respectively. The data in **Figure 4A** demonstrate that NF-κB-dependent gene expression did not change after a 5 Gy IR dose and increased remarkably only after exposure to a 15 Gy dose in all the reporter cell lines except STING-knockout cells. It is noteworthy that STING knockout sharply reduced NF-κB -dependent expression, while in the MAVS-knockout cells, on the contrary, this parameter strongly increased at 15 Gy, suggesting a dependence of NF-κB activation primarily on the STING pathway in irradiated monocytes. Analysis of luciferase expression that reflects the level of IFN-I expression and, at the same time, IFN-I-induced JAK/STAT signaling, shows a gradual increase of this value in THP1-Dual cells upon IR, with a twofold reduction of this parameter in IFNAR2-knockout cells and a threefold reduction in STING-knockout cells (**Figure 4B**). Interestingly, in THP1-Dual KO-STING, the highest level of IFN-I activity is detected after exposure to 5 Gy dose of IR. The most intriguing result was observed in MAVS-knockout cells – we could see a very high basic level of luciferase activity in the unexposed cells, and a twofold increase of this parameter in both 5 Gy- and 15 Gy-irradiated cells. This suggests that MAVS pathway when active can downregulate STING and thereby STING-induced expression of NF-κB- and especially IRF3-dependent genes.

To test this hypothesis, we indirectly assessed the activity of the transcription factors NF-κB and IRF3. Exposure to 5 and 15 Gy IR doses increased both the expression of IRF3 and p65 subunit of NF-κB, as well as the phosphorylation of Ser536 in the p65 subunit in THP1-Dual cells, indicating activity of this protein. In MAVS-knockout cells we observed downregulation of IRF3 expression upon IR, but increased expression and phosphorylation of p65. Finally, we could see an opposite trend in STING-knockout cells – increase of IRF3 upon IR and a remarkably reduced level of NF-κB p65 expression and especially phosphorylation (**Figure 4C**). While STING predictably was not expressed in the KO-STING cell line, the expression level of STING was much higher in MAVS-knockout cells compared to wild-type THP1, whereas MAVS expression did not change in STING-knockout cells as compared to wild-type cells.

In order to decipher how the MAVS and STING pathways impact the expression of particular interferon-stimulated genes depending on the applied IR doses, we examined ISG expression profiles in the same cell lines using PCR array. While the IFNAR2 knockout, which leads to impaired IFN-I-induced JAK/STAT signaling, predictably led to a reduced expression of most ISGs, the KO-MAVS and KO-STING cells displayed different expression profiles for different ISGs. Interestingly, we could outline the cluster of genes that are highly upregulated in KO-MAVS and downregulated in KO-STING cells, and another smaller cluster of ISGs, which are upregulated in KO-STING and downregulated or not changed in KO-MAVS cells even when the cells are not stimulated by IR (**Figure 4D**, **Supplementary Table 2**). This suggests that these basic signaling pathways are essential for the cytokine spectra of unstimulated monocytes, but differences in expression of “MAVS-dependent” and “STING-dependent” ISGs are increased upon stimulation of these pathways by the respective nucleic acid products elevated after IR.

**Figure 5:**
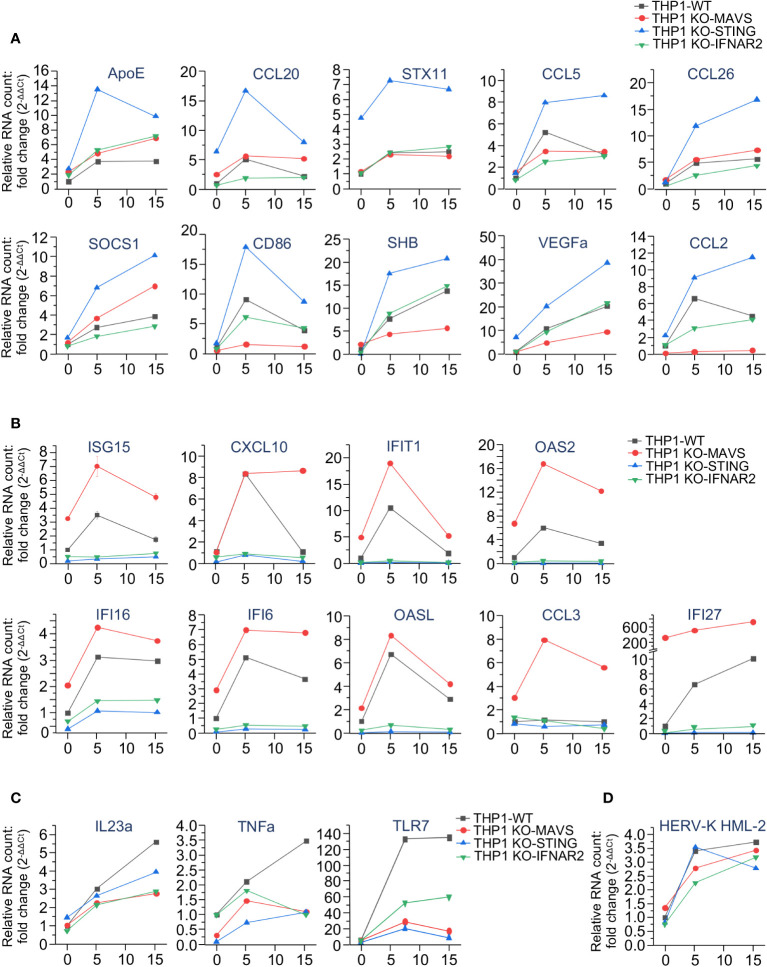
The expression of interferon-stimulated genes is differently regulated by MAVS- and STING-mediated signaling pathways upon irradiation. **(A-C)** Quantitation of expression of selected ISGs, upregulated in THP1-Dual KO-STING cells **(A)**, THP1-Dual KO-MAVS cells **(B)**, and downregulated in both THP1-Dual KO-STING and THP1-Dual KO-MAVS cells **(C)** upon irradiation. **(D)** Expression of HERV-K HML-2 *env* gene in the same cell lines after exposure to the indicated γIR doses. In all samples the gene expression is analyzed by RT-qPCR of total RNA, 48 h after exposure to γIR. The RNA level was measured by RT-qPCR vs. actin, GAPDH and GUSB reference housekeeping genes; error bars indicate SE of three independent measurements; paired *t* test.

To test this hypothesis, we then separately assessed the expression of selected genes that displayed a remarkable correlation with the activity of MAVS- or STING-mediated pathways after IR exposure. The results shown in **Figure 5** confirm our initial observations. Expression of ten ISGs demonstrated dramatic upregulation in STING-knockout cells. However, while some genes, such as ApoE, CCL5, CCL20, CCL26, STX11, and SOCS1 were significantly upregulated in STING-knockout cells, their expression was not or only slightly changed in KO-MAVs cells (**Figure 5A**), suggesting that some STING pathway-activated factors inhibited their expression, but they do not depend on the MAVS signaling. Other genes within this cluster (CD86, SHB, VEGFa, and CCL2) were also upregulated in KO-STING cells, but knockout of MAVS strongly downregulated these genes, indicating that their expression depends on MAVS signaling and is probably triggered by the transcription factor IRF3. Another ISG cluster displayed remarkable upregulation in the MAVS-knockout cells along with a dramatic reduction in expression in the KO-STING cell line (**Figure 5B**). Expression of these genes seem inhibited by any MAVS pathway-induced factors. At the same time, their expression is strongly upregulated by STING signaling, likely via NF-κB -dependent transcription and IFN-I-induced JAK/STAT pathway activation. Only a few genes, IL23a, TNFa, and TLR7, were downregulated upon knockout of both STING and MAVS signaling pathways (**Figure 5C**). Finally, the expression of HERV-K HML-2, which we previously found important for triggering MDA5/MAVS signaling pathway upon IR (28), was detected equally activated in all cell lines after radiation exposure (**Figure 5D**).

To compare our data with the effect of IR on the STING-dependent ISG expression in a different cell type, we re-analyzed a publicly available RNA-Seq dataset (GSE147085) with wild-type and STING-knockout FaDu human head and neck squamous cell carcinoma cells, exposed to 4 fractions of 2 Gy doses of X-ray (67) using TEcount software (68, 69) (**Supplementary Tables 3, 4**). While these transcriptomic data were obtained from epithelial cells, an identical cluster of radiation-induced genes, significantly downregulated in STING-deficient cells, was outlined (**Supplementary Figure 5**). Interestingly, sixteen out of twenty-one genes identified in this cluster were the same as in THP1 monocytes, including the highly upregulated genes ISG15, CXCL10, IFIT2 and 3, OAS2, IFI16 and DDX58. Since the FaDu cells were exposed to a fractionated IR, which mimics the treatment applied to radiation therapy patients, the observed results suggest that the nucleic acid sensing pathways are activated in a similar manner upon both single or fractionated IR doses.

Collectively, acquired data suggest that expression of many ISGs is regulated differently by RNA- and DNA-sensing signaling pathways and are likely dependent on the transcription factors that are activated by these pathways. Since, depending on the applied radiation dose, presence of dsRNA, RNA:DNA duplexes, and dsDNA, available for sensing by RLR or cGAS and IFI16 is different, exposure of monocytes to different IR doses can eventually activate the expression of various ISGs through either MAVS- or STING-mediated pathways.

### The response of monocytes to different radiation doses depends on the expression and intracellular localization of the components of RNA- and DNA-induced signaling pathways

**Figure 6:**
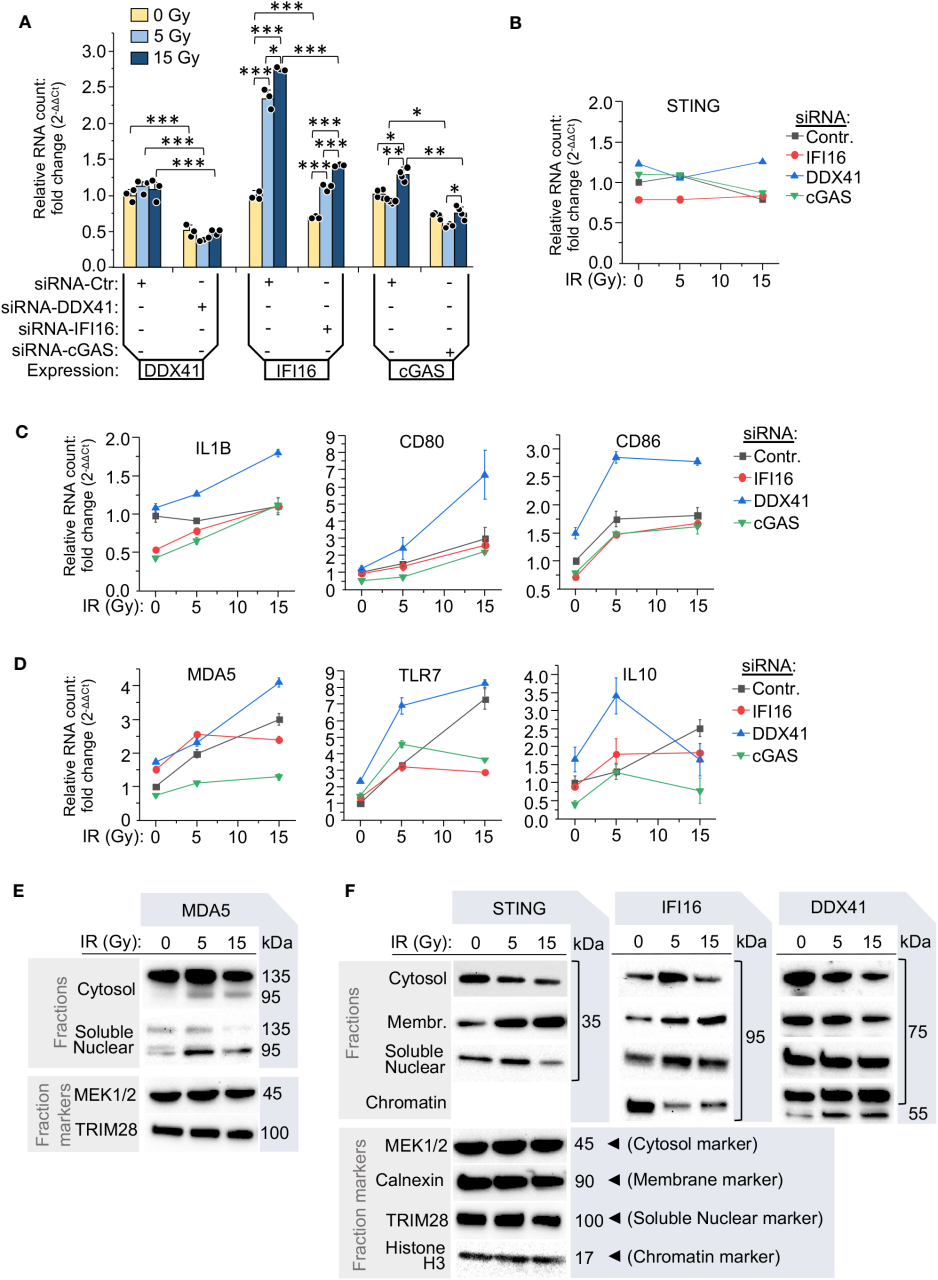
The components of RNA- and DNA-induced signaling pathways determine the inflammatory response of monocytes to various radiation doses. **(A)** Relative RNA count of DDX41, IFI16 and cGAS in THP1 cells transfected with either scramble siRNA (siRNA-Ctr) or the test siRNAs targeting DDX41, IFI16 or cGAS. The cells were cultured 24 h after transfection, exposed to the indicated IR doses and then cultured for additional 24 h. The RNA level was measured by RT-qPCR vs. GAPDH and GUSB reference housekeeping genes; n = 3 is shown. * *p*<0.05, ** *p*<0.01, *** *p*<0.001, paired *t* test. **(B-D)** Expression of STING **(B)**, markers of pro-inflammatory polarization of monocytes and macrophages, IL-1β, CD80 and CD86 **(C)**, and highly radiation-responded ISGs: MDA5, TLR7, and IL10 **(D)**. The cells were treated and analyzed as described in **(A)**. Error bars: ± SD of three independent biological replicates. **(E)** Immunoblotting of intracellular RNA sensor MDA-5 in the cytosolic fraction (vs. MEK1/2), and soluble nuclear fraction (vs. TRIM28) of THP1 cells, exposed to indicated doses of γIR, 48 h after exposure. Samples of 80 μg (cellular fractions) of total protein were used for gel loading. **(F)** Immunoblotting of DNA-induced pathway adaptor STING and functionally-associated DNA or RNA:DNA duplex interacting proteins IFI16 and DDX41 in the cytosolic (vs. MEK1/2), intracellular membrane (vs. calnexin), soluble nuclear (vs. TRIM28), and chromatin fractions (vs. histone H3) in THP1 cells, exposed to indicated doses of γIR, 48 h after exposure. Samples of 80 μg (cellular fractions) of total protein were used for gel loading. The protein loading controls of the fractions for the panels comprised by STING, IFI16 and DDX41 are the same, since proteins were separated in the same gel electrophoresis.

Since we found an increase of cGAS, IFI16 and STING in monocytes after IR exposure, along with the opposite trend shown by the protein DDX41, we analyzed the impact of these critical DNA-binding proteins on the interferon pathway and the expression of inflammation-related genes in monocytes after various doses of γIR. While siRNA-mediated silencing of DDX41, IFI16 and cGAS reduced their expression (**Figure 6A**), STING expression was stable and did not change on the RNA level upon knockdown of these genes and various IR doses (**Figure 6B**).

Markers of IFN-I signaling and monocyte/macrophage activation were elevated following exposure to IR in THP1 cells transfected with control siRNA. Knockdown of the initiating factors of the STING signaling pathway, cGAS and IFI16, revealed two groups among the tested genes in the context of their response to IR. The first group includes activation markers of pro-inflammatory macrophages, IL1B, CD80, and CD86, which are weakly dependent on the expression level of IFI16 and cGAS and were gradually upregulated in response to IR doses (**Figure 6C**). The second group included the RNA receptors MDA5 and TLR7, and the anti-inflammatory cytokine IL10. These ISGs were found downregulated in both the cGAS- and IFI16-silenced cells, but only after exposure to a 15 Gy dose (**Figure 6D**). IFI16 knockdown did not have a negative effect on their expression when the cells were exposed to a 5 Gy dose. On the contrary, these genes were upregulated, consistent with the previous data that upon medium radiation pressure, expression of these genes is regulated by the MAVS pathway. The most significant elevation of expression of both groups of genes was observed in the cells with knockdown of the helicase DDX41 (blue line in **Figures 6C, D**). On the RNA level, their expression increased by two- to nine-fold in the irradiated cells. For most genes, with exception to IL10, the highest level was observed after a dose of 15 Gy, suggesting that a decrease in DDX41 activity led to hyperactivation of the STING pathway, IFN-I expression and thereby upregulation of ISGs. Moreover, there was a very high level of CD86, TLR7 and IL10 RNA, the genes whose expression was upregulated predominantly by MAVS signaling, after a 5 Gy dose in the cells with silenced DDX41. This suggests that this RNA helicase might also be involved in the modulation of MAVS-mediated pathway.

Despite the fact that RIG-I and MDA5 are major cytosolic RNA sensors, recent publications indicate that RIG-I can translocate into the nucleus upon DNA damage conditions (70, 71). The proteins cGAS and IFI16 have also been found in the nuclear compartment either in association with foreign DNA or upon disruption of transcription machinery (72–75). To test whether the IR affects intracellular localization and integrity of the nucleic acid sensor molecules and likely the downstream signaling pathways, we analyzed the presence of these proteins in different cellular fractions. First, besides the increased amount of MDA5 in the cytosol after 5 Gy IR and the partial cleavage of this protein in irradiated cells, resulting in the truncated, 95 kDa form of MDA5 without a CARD domain (76), the doses of 5 and 15 Gy led to the detection of this truncated form also in the soluble nuclear fraction (**Figure 6E**). Second, both IFI16 and STING were revealed in the soluble nuclear fraction, especially after a 5 Gy IR dose; IFI16 was also abundant in this fraction after a 15 Gy dose (**Figure 6F**), suggesting an association of the localization of this protein with DNA damage. However, we also detected a rising amount of IFI16 in the fraction of intracellular membranes proportionally to the applied IR dose that correlated with the same trend for STING protein (**Figure 6F**). Finally, the RNA helicase DDX41 displayed an opposite trend and was decreased in the cytosolic and intracellular membranous fractions upon raising doses of IR, while in both the soluble nuclear fraction and chromatin, this protein was detected on a constantly high level independently of the applied dose of IR (**Figure 6F**). We provide a functional interpretation of these data below in the Discussion.

### RNA:DNA duplexes enhance both MAVS- and STING-mediated signaling and pro-inflammatory activation of monocytes

**Figure 7:**
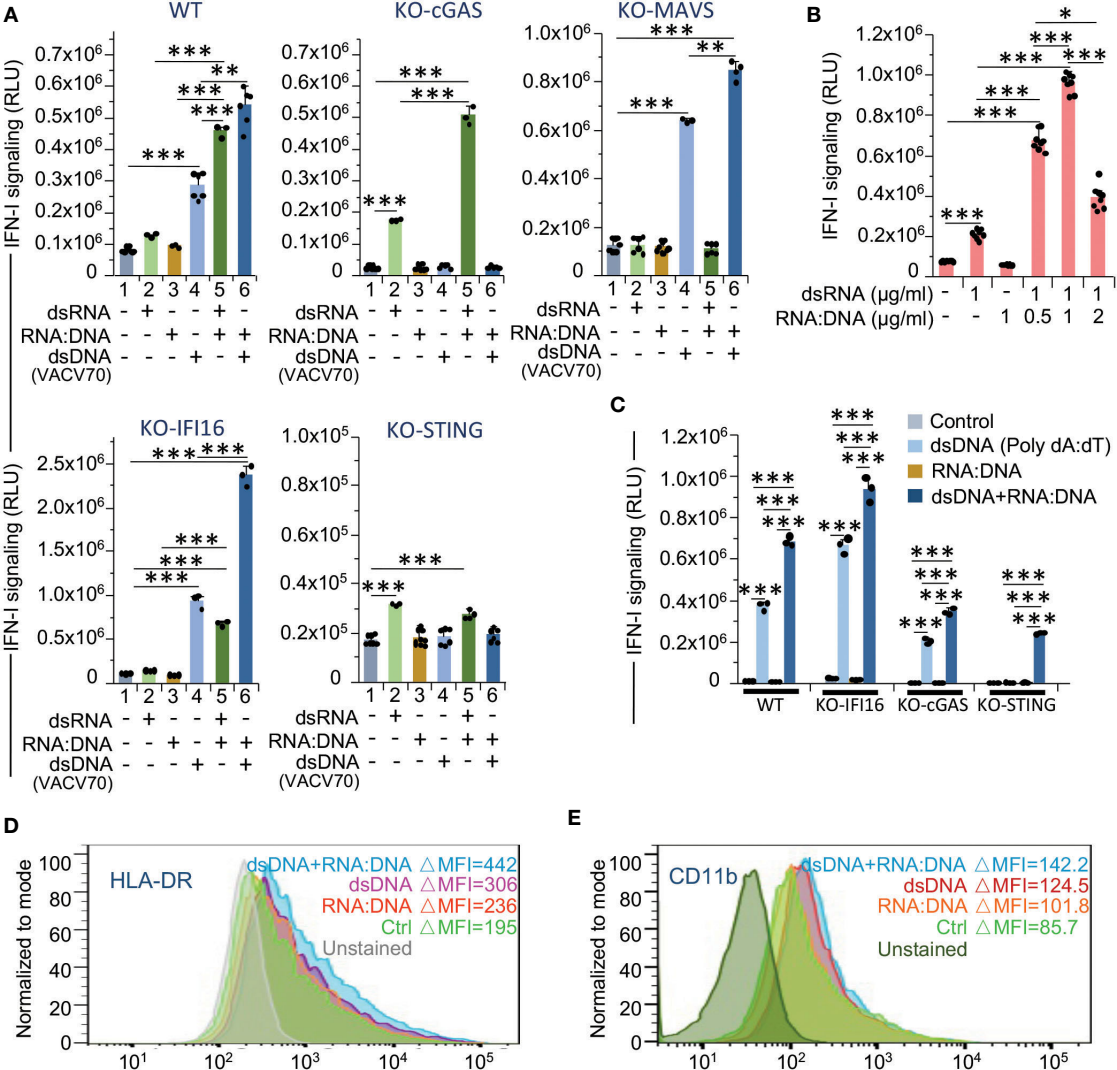
RNA:DNA duplexes contribute to both MAVS- and STING-mediated type I interferon signaling and pro-inflammatory activation of monocytes. **(A)** Activation of type I interferon-induced IFNAR signaling in untransfected cells and cells transfected with dsRNA, dsDNA or RNA:DNA duplex, or co-transfected with dsRNA + RNA:DNA duplex or dsDNA + RNA:DNA duplex using THP1-Dual cells (WT), THP1-Dual KO-cGAS, THP1-Dual KO-MAVS, THP1-Dual KO-IFI16, and THP1-Dual KO-STING cells. Parental THP1-Dual cells and the derived knockout cell lines express a secreted Lucia luciferase gene under the control of an IFNAR signaling-activated ISG54 minimal promoter as described in the legend for **Figures 4A, B**. After 48 h of incubation, staining with QUANTI-Luc substrate and measurement of luminescence was performed for each sample. Data were normalized to the untransfected controls. Error bars indicate ± SD of a minimum of 3 independent biological replicates. **(B)** Type I interferon-induced IFNAR signaling (Quanti-Luc substrate) in THP1-Dual cells either transfected with dsRNA or co-transfected with dsRNA and RNA:DNA duplex in a ratio of 2:1, 1:1 and 1:2. Data were collected 48 h after transfection; Error bars indicate ± SD of a minimum of 3 independent biological replicates. **(C)** Type I interferon-induced IFNAR signaling (Quanti-Luc substrate) in wild-type (WT) THP1-Dual cells and the indicated knockout cell lines either transfected with dsDNA poly(dA:dT) or co-transfected with poly(dA:dT) and RNA:DNA duplex in a ratio of 1:1. Data were collected 24 h after transfection; Error bars indicate ± SD of a 3 independent replicates. In panels A to C *** *p ≤* 0.001, ** *p ≤* 0.01, * *p ≤* 0.05; paired *t* test. **(D, E)** Percent of activated (HLA-DR^high^) **(D)** and CD11b^high^ **(E)** subsets within the populations of THP1 cells transfected with dsDNA, RNA:DNA duplex or co-transfected with dsDNA + RNA:DNA duplex, 72 h post-transfection. Only viable (DAPI-negative) single cells were analyzed.

To clarify the role of dsRNA, dsDNA and RNA:DNA hybrid ligands in the interferon response to γIR doses, we analyzed IFN-I signaling (luciferase activity) in THP1-Dual cells upon transfection with synthetic dsRNA (low-weight poly(I:C)), dsDNA (VACV70), or VACV70 RNA:DNA heteroduplex. While the transfection of wild-type THP1 cells with dsRNA and dsDNA predictably increased IFN-I expression/signaling, transfection with RNA:DNA hybrid did not affect IFN-I activity (**Figure 7A**). However, when cells were co-transfected with RNA:DNA duplex and dsDNA or dsRNA, in both samples we detected a drastic increase in interferon signaling. To test whether RNA:DNA heteroduplex activates IFN-I expression via enhancement of STING signaling pathway, as we hypothesized above, or contributes to both STING and MAVS pathways, we repeated the experiment with THP1-Dual cells with knocked-out expression of the either cGAS,IFI16, STING or MAVS. First, the knockout of cGAS gene dramatically reduced DNA-induced signaling, but did not affect dsRNA signaling. Notably, there was a threefold signal amplification upon co-transfection of KO-STING cells with dsRNA and RNA:DNA hybrid. This suggests that RNA:DNA heteroduplex may directly impact the MAVS pathway. This statement was confirmed by the experiment with KO-MAVS cells where we did not observe any effect of dsRNA or dsRNA+RNA:DNA co-transfection on the IFN-I signaling. Second, IFI16 knockout, while it did not affect MAVS signaling, dramatically enhanced dsDNA signaling as well as the impact of RNA:DNA heteroduplex, resulting in a threefold increase in IFN-I activity (**Figure 7A**, bottom line). Finally, STING knockout led to a dramatic reduction of both dsRNA- and dsDNA-induced signaling, along with the effect of RNA:DNA hybrid, which is consistent to the data on ISG expression (**Figure 4D**). The gene expression data also revealed a group of genes that were significantly upregulated upon knockout of MAVS (**Figure 5B**). These data can be considered to be consistent with the results of IFN-I activity analysis in KO-MAVS cells, indicating that this value was doubled in MAVS-knockout cells upon activation by dsDNA, as compared with wild-type cells (**Figure 7A**).

Since we observed the positive effect of RNA:DNA heterodimer on dsRNA signaling, we then tested whether this effect is concentration-dependent. Co-transfection of wild-type cells with dsRNA and RNA:DNA in different ratios indicated that the maximum enhancement of IFN-I activity was reached upon equal doses of both substrates (**Figure 7B**).

Initially, to study the effect of dsDNA on IFN-I signaling, we used the VACV70 dsDNA substrate, a synthetic oligonucleotide containing a 70 bp vaccinia virus DNA motif. To test whether the action of dsDNA sensors is different depending on the specific DNA substrate, we transfected the THP1-Dual cells with commonly used poly(dA:dT) synthetic dsDNA, a known activator of several cytosolic DNA sensors, such as cGAS, AIM2, DAI, DDX41, IFI16, and LRRFIP1 (77). Surprisingly, the IFI16-knockout cells also displayed a higher level of IFN-I activity than the wild-type cells, whereas cGAS knockout reduced this value (**Figure 7C**). Meanwhile, knockout of cGAS did not abrogate the interferon response to poly(dA:dT) treatment, likely due to involvement of alternative pathways, such as RIG-I/MAVS signaling, that was previously shown to be activated by poly(dA:dT) transcription products (78, 79). Co-treatment with RNA:DNA duplex increased this response and, moreover, led to detectable IFN-I activity even in STING-knockout cells, also suggesting the activation of STING-independent pathways.

To test whether the activation of IFN-I signaling cascade by dsDNA and RNA:DNA ligands contributes to pro-inflammatory activation and differentiation of monocytes, we then measured the presence of HLA-DR and CD11b markers on the surface of wild-type THP1 cells transfected with dsDNA (VACV70), or/and VACV70 RNA:DNA heteroduplex. In fact, treatment of cells with dsDNA and RNA:DNA hybrid together resulted in the highest level of HLA-DR (**Figure 7D**). Accordantly, the macrophage marker CD11b was also mostly elevated in these cells (**Figure 7E**).

## Discussion

In the present study we demonstrate that the response of monocytes to ionizing radiation is dose-dependent and is associated with elevated transcription, DNA damage and related signaling pathways that drive the expression of both type I interferons and interferon-stimulated genes, in addition to NF-κB-dependent genes involved in the pro-inflammatory activation of the cells. Of course, an increase in the applied dose of IR leads to raising cellular damage and, therefore, cell death and the release of DAMPs that affect viable monocytes and macrophages via pattern recognition receptors, such as TLRs, and activate their inflammatory response (5, 17, 21). This signaling is mediated primarily through NF-κB, a major regulator of the transcription of inflammation-related genes (80–82). However, the direct impact of IR on the cells, especially on radiation-resistant immune cells, such as monocytes and macrophages, is important for modulation of their activity, differentiation and inflammatory response. In this study, we were faced with the task of determining the effect of various IR doses on monocytes through the modulation of transcription and DNA damage and, therefore, the impact of intracellular RNA and DNA on the activation of these cells via specific nucleic acid sensors and downstream pathways, important for the innate immune response.

Earlier we showed that in monocyte-derived macrophages, medium doses of γIR (1-to-5 Gy) activated transcription of LTR retroelements in both sense and antisense directions, whose dsRNA triggered MDA-5/MAVS-mediated IFN-I expression and therefore ISGs via the JAK/STAT signaling pathway (28). Here we found that while this particular dose of γIR led to increased accumulation of dsRNA, higher doses (15 Gy) resulted in an increased number of dsDNA fragments and RNA:DNA heteroduplexes, the complex molecules which are the products of IR-induced DNA damage and disrupted transcription machinery. It was found that, along with dsDNA, RNA:DNA hybrids are capable of activating the STING signaling pathway (74, 83). According to our data, MAVS signaling is also activated in the presence of RNA:DNA hybrids, which leads to increased expression of type I interferons and proinflammatory modulators. Interestingly, the heteroduplex itself did not activate neither MAVS- nor STING-mediated pathway, but enhanced both of them when RNA or DNA ligands are present, respectively. This suggests that radiation-related accumulation of RNA:DNA hybrid molecules may upregulate both nucleic acid-induced pathways. However, since the accumulation of RNA substrates was found decreased upon high doses of IR, the RNA:DNA duplex molecules would likely primarily facilitate STING-mediated signaling when IR is increased beyond a certain threshold.

Overall, in monocytes, we identified different roles of the RNA sensing MAVS-mediated pathway and the DNA-sensing STING-mediated pathway in the expression of ISGs and other modulators of the inflammatory response. The MAVS-mediated signaling pathway was found to be involved primarily in the activation of IFN-I and ISGs upon medium radiation doses (1-7.5 Gy), whereas the STING pathway predominantly activated genes containing interferon-sensitive response element (ISRE) and NF-κB-binding sites in their promoters after high γIR doses. While some of these genes were expressed regardless of which signaling mechanism initially induced IFN-I production, we identified multiple genes whose expression changed depending on whether MAVS or STING signaling was activated or inhibited.

Identification of the cluster of ISGs that is highly upregulated in the MAVS-knockout cells where this critical RNA-sensing pathway is inhibited (TLR3 pathway may still be active), and elevated level of STING in these cells suggest that some MAVS pathway-induced ISG products can inhibit STING expression and signaling. Indeed, a recent study by Ghosh and co-authors (84) showed that one of the ISGs, Interferon-Inducible Human Oligoadenylate Synthetase-Like (OASL), enhances RIG-I-mediated IFN-I induction upon RNA virus infection, but inhibits STING pathway-induced IFN-I expression via direct and specific binding to cGAS independently of dsDNA. In the samples we have analyzed, expression of OASL in the MAVS-knockout cells was detected on the same level as in the wild-type THP1, and inhibited in the THP1 KO-STING cell line, suggesting that in irradiated monocytes, expression of this gene is dependent on STING signaling as well. However, among the genes that are dramatically upregulated in the STING-knockout cells, we identified Suppressor Of Cytokine Signaling 1 (SOCS1), a component that participates in a negative feedback loop to attenuate type I and type II interferon signaling via the JAK/STAT pathway (85). Since this factor is upregulated in varying degrees by IR in both STING- and MAVS-knockout cells, we speculate that it may suppress IFN-I signaling by inhibiting both RNA- and DNA-sensing pathways, but downregulates STING signaling in the first place.

Analysis of the effect of IR on STING-knockout and wild-type head and neck squamous cell carcinoma cells, performed by Hayman and colleagues (67), revealed the role of STING as an intrinsic regulator of survival for tumor cells following exposure to IR. The authors showed that STING regulates transcription of the genes that control ROS generation, specifically ISG15, which is involved in the regulation of reactive oxygen homeostasis. Thus, activation of the STING pathway upon DNA damage led to an elevated level of ROS in irradiated tumor cells and facilitated cell death, whereas STING-knockout cells demonstrated significantly enhanced radiation-resistance. We also identified ISG15 among the genes upregulated by IR, especially when the MAVS pathway is inhibited. Since elevation of ROS is thought to be a critical factor in the regulation of the pro-inflammatory macrophage phenotype (86–89), and these cells are themselves very resistant to high levels of ROS and nitric oxide (90, 91), we suggest that a STING pathway-mediated increase of ROS concentration is an additional factor that contributes to the differentiation of monocytes towards pro-inflammatory macrophages after high doses of IR.

An approximately one-day delay in the expression of RNA and DNA sensors and components of their pathways was clearly observed in THP1 cells and partially in primary monocytes. This was probably due to the accumulation of RNA and/or DNA and RNA:DNA substrates recognized by the RNA- and DNA-sensors in the cytoplasm of irradiated monocytes, which during this time underwent changes associated with differentiation to the pro-inflammatory phenotype. Elevated transcription of dsRNA, that is likely derived to a large extent from retroelements, detected after exposure to medium IR doses, might be the result of single- and double-stranded DNA breaks (SSBs and DSBs respectively) and DNA repair activity that leads to active DNA demethylation in the adjacent areas and therefore activation of aberrant transcription. Interestingly, a recent study by Wu and coauthors showed that in post-mitotic neurons, the single-strand breaks occurred in specific areas of the genome located within enhancers at or near CpG dinucleotides and sites of DNA demethylation could lead to aberrant gene expression (92).

Various forms of dsRNA, accumulated in the cytoplasm, as well as 5′-triphosphorylated ssRNA bind to the RLR receptors MDA5 and RIG-I respectively (57). Upon RNA binding, they are activated via oligomerization through both RNA- and polyubiquitin-dependent mechanisms and associate with MAVS, localized on the mitochondria (93). This mechanism initiates downstream signaling that results in IFN-I expression and secretion. Meanwhile, recent publications indicated that RIG-I can translocate into the nucleus upon viral infection or after genomic DNA damage (70, 71). Earlier studies showed that the truncated, 95 kDa form of MDA-5 without a CARD domain was also detected in the nuclei upon apoptosis and accelerated Fas ligand-mediated DNA degradation (76). Guo and coauthors (71) demonstrated that the nuclear form of RIG-I is recruited to DSBs and suppresses non-homologous end joining via interaction with XRCC4, one of the key factors involved in DSB repair, thereby counteracting DNA damage repair. The elevated level of truncated MDA5 in the nuclear fraction of irradiated monocytes, detected in our experiments, suggests that upon IR, this RLR protein can also accumulate in the nucleus upon DNA damage and probably contribute to the accumulation of DNA fragments and probably RNA:DNA hybrids which, in turn, activate an inflammatory response via the STING-mediated pathway.

cGAS is a key DNA sensor that directly binds to dsDNA, leading to the synthesis of 2’,3’-cGAMP, a secondary messenger that binds to and activates STING, thereby triggering IFN-I production. In our experiments, cGAS deficiency led to a dramatic reduction of dsDNA-induced IFN-I signaling and decreased expression of the tested ISGs and pro-inflammatory markers, especially upon high IR doses. According to earlier reports (74, 94), cGAS also interacts with RNA:DNA duplexes, that in turn triggers the STING pathway. Our results indicated an essential role of RNA:DNA hybrids in the enhancement of DNA-induced STING signaling, whereas heteroduplex molecules themselves did not exert significant effect on this pathway. The mechanism of this effect currently requires further investigation.

We also detected a remarkable increase of Interferon Gamma Inducible Protein 16 (IFI16) after the radiation exposure. IFI16 is known to cooperate with cGAS and amplifies its function during DNA sensing for full activation of the STING signaling pathway (95). In human macrophages, cGAS has also been shown to be involved in downstream STING signaling through the recruitment and activation of TBK1 in the STING complex (96). Published data indicate that IFI16 senses DNA in both cytoplasmic and nuclear compartments (97, 98). Indeed, Dunphy and co-authors (61) showed that after DNA damage, IFI16 within an intranuclear multimolecular complex is activated by DNA fragments and then, upon export to the cytoplasm, induces non-canonical STING signaling that results in NF-κB activation. The proteins cGAS and IFI16 have been found in nuclear compartment in association with foreign DNA or with RNA:DNA duplexes upon disruption of transcription machinery (72–75). We also detected a stably high level of IFI16 in the soluble nuclear fraction of irradiated THP1 monocytes. However, in unirradiated cells, knockout of IFI16 resulted in increased STING-mediated signaling induced by both dsDNA and by the combination of dsDNA and RNA:DNA duplexes. In contrast, after IR, we observed the negative effect of IFI16 knockdown on the expression of inflammation markers and IL10. Increased amount of both IFI16 and STING in the fraction of intracellular membranes proportionally to the applied IR dose suggests, that in irradiated cells IFI16 mediates STING signaling triggered by either cytoplasmic or nuclear DNA fragments (as described in (25, 66)) or by RNA:DNA hybrids (65). Whereas in unirradiated monocytes, the function of this DNA sensor can be more diverse in the tuning of the STING pathway to avoid hyperactivation of the interferon response upon excess abundance of nucleic acid substrates.

DNA damage, either SSBs or DSBs, create free 3’-DNA ends that promote the annealing of RNA to DNA to form hybrids, originating from both hybridization with existing nascent RNA or *de novo* transcripts (99, 100). These specific three-stranded nucleic acid structures that consist of an RNA:DNA hybrid and a non-template DNA strand are called R-loops and have been shown to be involved in triggering NF-κB-mediated inflammation via activation of the cGAS/STING signaling pathway (74, 94). Drastic activation of IFN-I signaling in response to co-transfection of cells with dsDNA and RNA:DNA heteroduplexes and an increased pool of RNA:DNA hybrids in irradiated monocytes, particularly after exposure to high doses of IR, that correlated with elevated STING-mediated IFN-I upregulation and ISG expression, suggest that R-loops may represent a significant source of immune activation for monocytes and MDMs in response to IR. Interestingly, upregulation of STING expression and phosphorylation of Ser366 in STING protein correlated with decreased expression of the DEAD Box helicase DDX41. Recent studies indicated that this protein can reduce R-loop accumulation by unwinding RNA-DNA hybrids and thereby limit cGAS/STING activity and the associated inflammatory response (74, 101). Earlier publications also showed that RNA:DNA hybrids generated during retroviral reverse transcription are sensed by both DDX41 and IFI16 proteins, which are involved in the control of the STING-mediated immune response to retroviral infection (64–66). Thus, a reduced level of DDX41 in irradiated monocytes could facilitate higher STING-mediated responses to the accumulating R-loops. Notably, DDX41 is abundant in nuclei upon all tested doses of IR. However, since STING had low abundance in the nuclear fraction, and should be localized in the ER membrane for its own signaling activity (102, 103), a reduced amount of DDX41, specifically in the cytosolic and especially intracellular membrane fractions upon IR, can explain the upregulation of STING pathway and hence the elevated IFN-I and inflammatory response.

Taken together, our data suggest that exposure of human monocytes to increasing doses of ionizing radiation leads to first, cytoplasmic accumulation of RNA and activation of the MAVS-mediated pathway, which is likely associated with IR-induced aberrant transcription activity This pathway results in upregulation of numerous genes that contain ISREs in their promoters, including the SASP-associated genes, such as CCL2 (104), CCL5 (105), and CCL20. Second, an increase in the radiation dose leads to a higher level of DNA damage and a related accumulation of dsDNA fragments and R-loops. Double-stranded DNA fragments activate STING signaling, primarily through the DNA sensor cGAS. RNA:DNA hybrids that accumulate following decreased expression or destruction of the helicase DDX41, which is known to be able to prevent the signaling activity of RNA:DNA duplexes, in turn facilitate the STING signaling pathway. While the STING-mediated pathway activates both IRF3- and NF-κB-dependent gene expression, severe DNA damage is also triggering apoptotic changes in irradiated cells that may contribute to elevated NF-κB-activated transcription and a transition of the monocytes towards pro-inflammatory stage. Overall, further investigation of radiation dose-related changes in gene expression and associated signaling mechanisms will facilitate a better understanding of the molecular machinery involved with the phenotypic transition of monocytes and macrophages. This might contribute to identification of potential new targets for therapeutic interventions aimed at mitigating altered inflammatory activity, managing acute and counteracting chronic inflammation after radiation, and biodosimetry tasks.

## Materials and methods

### Resources and reagents

Information on resources and reagents used in this study is shown in **Supplementary Table 5**. This study did not generate new unique reagents.

### Cells

The acute monocytic leukemia cell line THP1 (ATCC) was maintained at 37°C and 5% CO_2_ in RPMI-1640 media supplemented with 10% FBS, penicillin/streptomycin, and L-Glutamine. The reporter cell lines THP1-Dual, THP1-Dual KO-IFNAR2, THP1-Dual KO-MAVS, THP1-Dual KO-STING, THP1-Dual KO-cGAS, and THP1-Dual KO-IFI16 (InvivoGen) were maintained at 37°C and 5% CO_2_ in RPMI-1640 media containing 25mM HEPES (Gibco), 10% heat inactivated FBS (FBS-HI, Quality Biological), 100µg/ml normocin (InvivoGen), penicillin/streptomycin and L-Glutamine according to manufacturer’s protocol. The samples of monocyte enriched fraction 5 of human PBMCs*, separated by elutriation (Terumo BCT Elutra Cell Separation System)*, were obtained from The NIH Blood Bank. Primary monocytes were irradiated or sham-irradiated and cultured in Dutch modified RPMI-1640 culture medium (Gibco) supplemented with 10% human serum (Corning), penicillin/streptomycin, sodium pyruvate and GlutaMAX (Thermo Fisher Scientific) for two to eight days. Half of the medium was replaced every third day.

### Gamma irradiation

The irradiation experiments were performed in a ^137^Cs irradiator at ambient temperature (22-25°C) and atmosphere. The dose rate during all experiments was approximately 32 Gy/h; however, the exact irradiation time was calculated on the day of each experiment based on the dose rate for the current date.

### Transfections

The Lipofectamine RNAiMAX reagent protocol (Thermo Fisher) was modified for THP1 and used to transfect the cells with siRNA. Briefly, cells were plated into fresh RPMI-1640 medium supplemented by 10% FBS-HI in concentration 0.5 x 10^6^ cells/ml and incubated for 24 h. After wash with PBS, THP1 cells were treated with 0.05% trypsin and 0.53 mM EDTA for 1 minute at 37°C and immediately washed with fresh RPMI-1640 plus 10% FBS-HI medium. Then 2 x 10^6^ cells were incubated with transfection complexes containing 30 pM siRNA and 9 µl Lipofectamine RNAiMAX in 500 µl of Opti-MEM medium (Thermo Fisher) in wells of 12-well tissue culture plates. The cells were incubated with siRNA–lipid complexes at 37°C and 5% CO_2_ for 5-6 h, after that the cells were precipitated by centrifugation at 200 x g for 5 min and treated with trypsin-EDTA solution as described above. Then the cells were washed with RPMI-1640 medium plus 10% FBS-HI and then with PBS. After wash, the cells were placed into 4 ml of complete culture medium (RPMI 1640 + 25mM HEPES+ 10% FBS-HI+1% penicillin/streptomycin+ 1% L-Glutamine) and incubated at 37°C and 5% CO_2_ in 25 cm^2^ cell culture flasks overnight. Then 1 x 10^6^ cells were placed into wells of 12-well tissue culture plates containing 1 ml of complete culture medium, exposed or not to IR and cultured for 48 h.

Transfection of THP1 and reporter THP1-Dual cells with the low molecular weight polyinosine-polycytidylic acid (LMW Poly(I:C)) with an average size 0.2-1.0 kb (InvivoGen), a 70 bp dsDNA containing the vaccinia virus DNA motif (VACV70) (InvivoGen), a repetitive synthetic double-stranded DNA poly(deoxyadenylic-deoxythymidylic) [poly(dA:dT)], and RNA:DNA hybrid with VACV70 homologous sequence was performed with cationic lipid-based transfection reagent LyoVec (InvivoGen). Hybridization of ssRNA and DNA VACV70 oligonucleotides was previously performed in annealing buffer, containing 10 mM Tris-HCl, pH7.5, 50 mM NaCl, and 1 mM EDTA using the following program: 95°C for 2 min, cool to 25°C for 45 min, 4°C for 15 min. The cells plated in fresh complete culture medium (see above) were transfected or co-transfected with LyoVec/nucleic acid complexes with the final concentration of 1 µg/ml nucleic acid and 6 µg/ml LyoVec, (1:6 ratio w/w) in 25 cm^2^ cell culture flasks or 24-well plates according to the manufacturer’s protocol, and then incubated in the same culture medium for 48-72 h at 37°C and 5% CO_2_.

### Flow cytometry

For the surface staining, human elutriated monocytes were washed with HBSS buffer (Gibco) supplemented with 2% FBS-HI and blocked with human Fc block (BD Biosciences) according to manufacturer’s instructions. Then, the cells were incubated for 30 min on ice with optimal concentration of anti-human antibodies in HBSS+2% FBS-HI buffer: HLA-DR-PECy5 (LN3), CD80-FITC (B7-1) (Thermo Fisher Scientific), CD11b-APC (D12), CD44-PE (515) (BD Biosciences), CD207-PECy7 (15–2), CD86-APC (BU63), CD45RA-PECy7 (HI100), CD14-APC (M5E2), CD16-PE (3G8), Lineage: CD19-FITC (HB19), CD66b-FITC (G10F5), CD20-FIT(2H7), CD3-FITC (HIT3a), CD56-FITC (MEM-188) (BioLegend). After staining, the cell pellets were washed with HBSS+2% FBS-HI, resuspended in 1 µM DAPI solution in HBSS+2% FBS-HI, filtered through nylon mesh and analyzed using LSRII or Symphony A5 flow cytometers (BD Biosciences). Data analysis was performed with the FlowJo (BD Biosciences) software. Fluorescent minus one (for multicolor staining) or DAPI-only (for single-color stained) controls were used to set up positive and negative populations gates. Doublets (on FSC-H vs. FSC-A dot plot), cell debris (gated out on side vs. forward scatter dot plot) and dead (DAPI^high^) cells were excluded from analysis.

To account for the increased autofluorescence of primary monocytes due to radiation-induced increased cell size and granularity, FMO controls, used to set up positive and negative gates for each staining, were composed of equal numbers of primary cells from the samples exposed to all radiation doses, where non-irradiated and irradiated cells were mixed in 1:5 ratio. Since background fluorescence of THP1 cells is essentially affected by increased size and granularity followed the raised doses of gamma-radiation and less affected upon nucleic acid transfection, we used delta MFI (median fluorescent intensity) parameter, i.e. the difference in median fluorescent intensity between the stained and unstained THP1 cells in the same channel of fluorescence.

### RNA isolation, reverse transcription and quantitative PCR analysis

Total RNA was extracted from cells using RNeasy spin columns (Qiagen) followed by on-column DNase digestion according to the manufacturer’s protocol. After resuspending in RNase-free water, a 500-to-2,000 ng of total RNA was used to generate cDNA with the High Capacity cDNA Reverse Transcription Kit (Thermo Fisher Scientific), 10U of RNase Inhibitor (Applied Biosystems) and either oligo-dT-primer or RT random hexamer transcription primers following the manufacturer’s protocol. The RT reaction was performed with and without reverse transcriptase in the reaction mixture to ensure genomic DNA removal for qPCR analysis. Quantitative real-time PCR was performed with 2 µl of minimum 2-fold diluted RT reaction mixtures, the SSO Advanced Universal SYBR Green Supermix (Bio-Rad) and the primers specific for particular genes (**Supplementary Table 5**). The optimal PCR program was as follows: 95°C for 3 min; then 95°C for 10 sec, 60°C (56-to-62°C depending on the optimal annealing temperature) for 40 sec and ran 41 cycle for both the tested and reference genes. Real-time PCR reactions were carried out at least in triplicate. Relative gene expression was determined by the _ΔΔ_Ct ratio method using Bio-Rad CFX Manager 3.1 software. The fold gene expression in all experiments was calculated in relation to GUSB and GAPDH as reference housekeeping genes. The PCR efficiencies for the primer sets were estimated to be 100 ± 10% by titration analysis (106).

### Pathway-focused gene expression analysis using PCR array

RNA was isolated from the cells as described above and subjected to reverse transcription reaction with random hexamer primers according to the manufacturer’s protocol (Thermo Fisher Scientific). Real-time PCR was performed in 96-well plates from RT^2^ Profiler PCR Arrays designed for human type I interferon-related genes or human cytokines & chemokines (Qiagen) with 1 µl of cDNA equivalent to 50 ng RNA for each reaction and SsoAdvanced Universal SYBR Green Supermix (Bio-Rad) according to the manufacturer’s protocol (Qiagen). Relative gene expression against the GAPDH and HPRT1 housekeeping genes was determined by the _ΔΔ_Ct ratio method and presented on scatter plots or heatmaps.

### Analysis of dsRNA, RNA-DNA hybrids and dsDNA by dot blot

RNA was isolated by Trizol reagent (Thermo Fisher Scientific) according to manufacturer’s protocol and solubilized in RNase, DNase-free water containing 0.1mM EDTA. DNA for the analysis of dsDNA and RNA:DNA hybrids was isolated by Wizard DNA Purification kit (Promega) according to manufacturer’s protocol without RNase A treatment and solubilized in TE buffer (10mM Tris-HCl, 1mM EDTA, pH 8.0) for 2h at 65°C on the thermo-mixer. For dsRNA and RNA:DNA heteroduplex analysis, the RNA and DNA concentrations were adjusted to 400ng/µl and 200ng/µl respectively using Implen NanoPhotometer NP80 and then twofold serial dilutions were prepared. For dsDNA analysis, DNA concentrations were normalized by MaqQuant Plus DNA kit V2 (MagBio) to 30ng/µl DNA in water, and then serial twofold dilutions were made in water. The aliquots of 2µl/dot of either RNA or DNA solutions were loaded on positively charged Nytran N45 nylon membrane (GE Healthcare/Whatman). The membranes were crosslinked in the UV Crosslinker (Fisher Scientific) for 2min at 120,000 µJ), blocked for 1 h at room temperature in TBS-Tween 0.05%+ 5% dry milk (except RNA-blotted membrane where blocking step was opt out), and incubated overnight at 4°C with gentle rotation in corresponding primary antibodies. The following primary antibodies were applied. 1) for dsRNA – undiluted pan-enterovirus 9D5 mouse IgG3 monoclonal antibody (EMD Millipore #EN0541). 2) for RNA:DNA duplexes – anti-RNA-DNA hybrid antibody (S9.6, mouse IgG2a, Kerafast # ENH001) diluted 1:1,000 in TBS-Tween 20 0.05% + 2.5% dry milk. 3) for dsDNA – anti-dsDNA antibody [3519 DNA] (Abcam #ab27156) diluted 1:5,000 in TBS-Tween 20 0.05%+2.5% milk. After the incubation with primary antibodies, the membranes were washed five times, 5 min each, in TBS-Tween 20 0.05%, and labeled with secondary HRP-conjugated antibodies (Cell Signaling, 1:2000 in TBST- 5% milk) for 1 h at room temperature with gentle rotation. After washing five times with TBST buffer, membranes were probed with Clarity One Chemiluminescent ECL substrate (Bio-Rad) and visualized using ChemiDoc MP Imaging System (Bio-Rad). To validate the specificity of S9.6 antibody for RNA:DNA duplexes, a 4,000ng nucleic acid extract aliquot from THP1 cells exposed to 5 Gy of γIR (48 h post-exposure) was incubated with or without RNase H (10U) in 1x RNase H buffer (New England Biolabs) for 1 h at 37°C. Reaction was stopped by heating mixtures at 65°C for 20 min, then the twofold serial dilutions were loaded onto Nytran membranes, crosslinked, blocked and incubated with S9.6 or dsDNA recognizing antibodies. All further steps were performed as described above.

### Detection of NF-κB-activation and IFN-I induction using THP1-dual cells

Reporter THP1-Dual, THP1-Dual KO-IFNAR2, THP1-Dual KO-MAVS and THP1-Dual KO-STING cells (InvivoGen) were cultured as described above. On the day of experiment, the cells were placed into Test-RPMI-1640 medium (without Normocin) and exposed to various γIR doses. After subsequent incubation of cells for 48h at 37°C and 5% CO_2_, the culture supernatants were harvested and subjected to analysis of activity of a secreted embryonic alkaline phosphatase (SEAP) (NF-κB-activated expression) and luciferase activity (IFNAR signaling) using GloMax Multi Detection System (Promega) according to the manufacturer protocol.

### Cell fractionation

Preparation of cytoplasmic, membrane, nuclear soluble and chromatin-bound protein fractions was performed using Subcellular Protein Fractionation Kit (Thermo Fisher Scientific) according to the manufacturer’s protocol. Then 20-40 µg of total protein from each fraction was taken for immunoblotting. The antibodies against MEK1/2 (D1A5), calnexin (C5C9), histone H3 (D1H2) (all from Cell Signaling), and TRIM28 (Proteintech) were applied to recognize respective host cellular proteins used as loading controls for the cytoplasmic, membrane, chromatin-bound and soluble nuclear fractions respectively.

### SDS-PAGE and immunoblotting

Whole-cell lysates in RIPA buffer or extracts of cell fractions in the specific fractionation buffer from Subcellular Protein Fractionation Kit (Thermo Fisher Scientific), mixed with 1x Halt protease inhibitor cocktail (Thermo Fisher Scientific) and phosphatase inhibitors (for phospho-proteins) were normalized by the total protein count using the Pierce BCA Protein Assay kit (Thermo Fisher Scientific). Normalized extracts were resuspended in Laemmli buffer with 5% β-mercaptoethanol (Bio-Rad), heated at 95°C for 5 min and spun down at 15,000 x g for 1 min. Equal amounts of protein (20-80 µg) were loaded onto 4-20% Tris-Glycine-SDS polyacrylamide gels. Proteins were transferred onto a PVDF membrane or 0.22 nm nitrocellulose membrane at 10 mA and 4°C overnight. Gels were stained with GelCode Blue Safe Stain (Thermo Fisher). Membranes were blocked with TBS containing 0.1% Tween-20 and 5% dry milk or 5% BSA (for phospho-proteins) and incubated overnight at 4°C with the appropriate primary antibody. Membranes were then washed 5 times, incubated with the respective HRP-conjugated secondary antibody (Cell Signaling), and after washes, with Clarity One chemiluminescence (ECL) reagent (Bio-Rad). Images were obtained in ChemiDoc MP Imaging System and analyzed by ImageLab software (Bio-Rad).

### Multiplex cytokine/chemokine immunoassay

Magnetic beads-based BioPlex Multiplex immunoassays (Bio-Rad) for human chemokine and cytokine detection were performed with THP1 cells’ filtered culture supernatants according to manufacturer instructions. Protein concentrations were determined using serial dilutions of protein standards. Data were acquired on Bio-Plex 200 (Bio-Rad) instrument and analyzed by Bio-Plex Manager v.6 Software.

### RNA-sequencing data acquisition and gene expression analysis

Publicly available raw or pre-processed FASTQ files were downloaded from the NCBI Gene Expression Omnibus repository using the prefetch command from the NCBI SRA Toolkit as.sra files and converted into FASTQ files with the fasterq-dump command. FASTQ files were adaptor clipped and quality trimmed with Trimmomatic. For paired-end sequencing datasets, only paired reads were kept for downstream analysis. The standard TEcount pipeline was followed to map reads to the genome using GRCh38.103 and count the number of mapped reads to normal genes (68). Read counts were imported into R and analyzed with edgeR to normalize the raw data and to determine the CPM, RPKM, and fold-change (when applicable) (107). The normalized and raw gene expression data are shown in the **Supplementary Tables** excel file.

### Quantification and statistical analysis

The details of the statistical analysis of experiments, including used statistical tests and number of replicates are provided in the figure legends. Statistical measurements and plotting were performed using OriginPro 2020 (OriginLab). All values in this study represent means of at least three biological replicates ± SD. Two-tailed paired *t* tests and two-way ANOVA were used to compare differences between two groups and multiple groups, respectively. For the datasets with a minimum of five biological replicates and heterogeneity of variance, a Wilcoxon signed rank test was performed for paired observations. Real-time PCR data were quantified and analyzed using BioRad CFX Manager 3.1. The option of Gene Study analysis was performed to assess gene expression data obtained using RT^2^ Qiagen PCR arrays.

## Data availability statement

The original contributions presented in the study are included in the article/**Supplementary Material**. Further inquiries can be directed to the corresponding author.

## Ethics statement

Ethical approval was not required for the studies on humans in accordance with the local legislation and institutional requirements because only commercially available established cell lines were used.

## Author contributions

NM designed and performed the majority of the experiments, analyzed results and wrote distinct sections of the manuscript. ER helped with the experiments, performed bioinformatic analysis and contributed to the discussions and writing the manuscript. SI conceived, designed and supervised the project and wrote the manuscript. All the authors helped with data analysis. All authors contributed to the article and approved the submitted version.
