# Supplementary material for: Cellular RNA and DNA sensing pathways are essential for the dose-dependent response of human monocytes to ionizing radiation
Source: Front Immunol. 2023 Dec 12;14:1235936. doi: 10.3389/fimmu.2023.1235936 (PMC10751912; doi:10.3389/fimmu.2023.1235936)
Supplement: Supplementary file 1 [file DataSheet_1.pdf]

Supplementary Material

Cellular RNA and DNA Sensing Pathways are Essential for the Dose-Dependent Response of Human Monocytes to Ionizing Radiation

Natallia Mikhalkevich, Eric Russ and Sergey Iordanskiy

1. Supplementary Figures

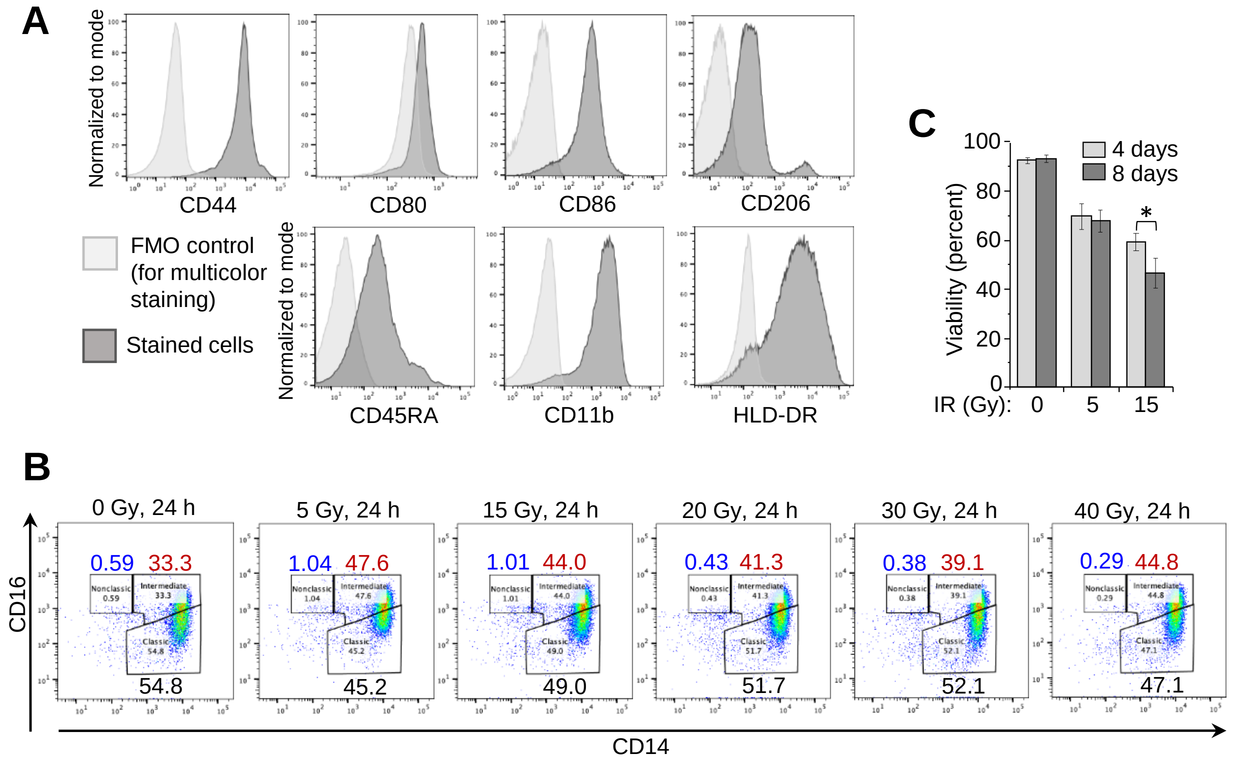

Subpopulations of monocytes: Black – classical, Red – intermediate, Blue – nonclassical

<u>Supplementary Figure 1</u>. Gamma radiation changes the population profile of primary monocytes and affects cell viability at late time points.

(**A**) Presentation of monocyte markers on the surface of elutriated primary human monocytes measured by flow cytometry with specific staining vs. background signal determined by Fluorescence Minus One (FMO) controls in viable (DAPI-) cells (graphs show representatives of 3 independent measurements). (**B**) Analysis of monocyte population in fraction 5 of elutriated human PBMCs profiled by flow cytometry 24 h after exposure to indicated doses of γIR, shown for 1 representative of 5 tested donors. Lineage markers were used to exclude contamination with lymphocyte subsets as described in Fig. 1A. Anti-CD14-APC and anti-CD16-PE coupled antibodies were used to outline subpopulations of classical, intermediate and non-classical monocytes. (**C**) Viability of THP1 cells measured by flow cytometry as a percentage of DAPI-negative cells in the total population, 4 and 8 days after exposure to indicated doses of γIR. Error bars indicate ±SD of three independent biological replicates; * *p*<0.01.

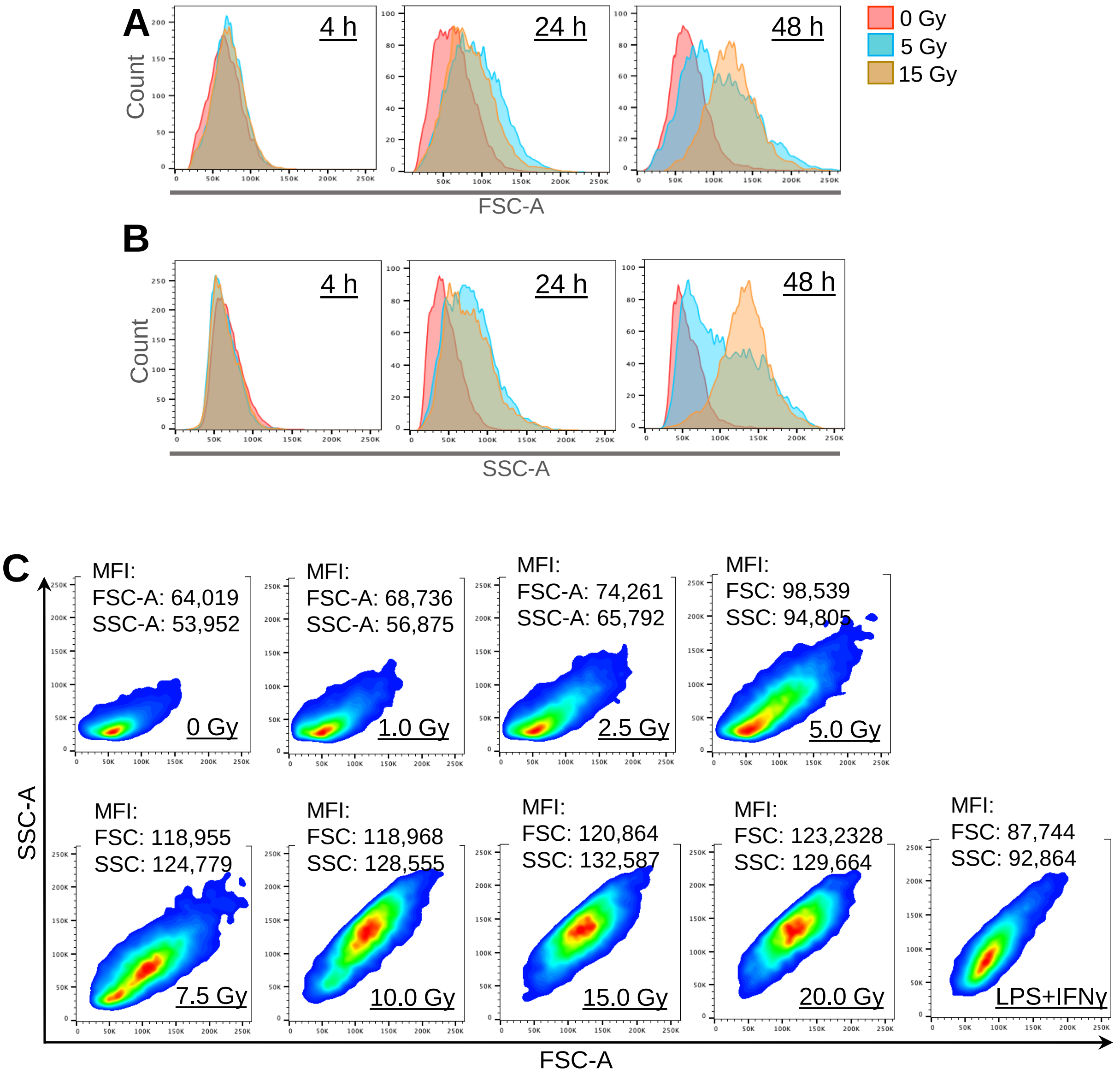

Supplementary Figure 2. Effect of gamma radiation doses on cyto-morphological parameters of human monocytic cell line THP1.

(**A**) Size distribution (FSC-A) of THP1 cells exposed to indicated γIR doses, detected by flow cytometry measurement of the fluorescence intensity of viable (DAPI-) cells; 4, 24, and 48 h post-IR. (**B**) Cell granularity distribution (SSC-A) of the same populations (described in (C)) of viable (DAPI-) THP1 cells. (**C**) Pseudocolor plots (Side scatter area: SSC-A – indirect measure of cell composition/granularity vs Forward scatter area FSC-A – indirect measurement of cells size) representing distribution of viable (DAPI-negative) THP1 cells exposed to indicated γIR doses, 48 h post-IR or treated with LPS (100 ng/mL) and interferon-gamma (25n/mL). MFI – median fluorescence intensity.

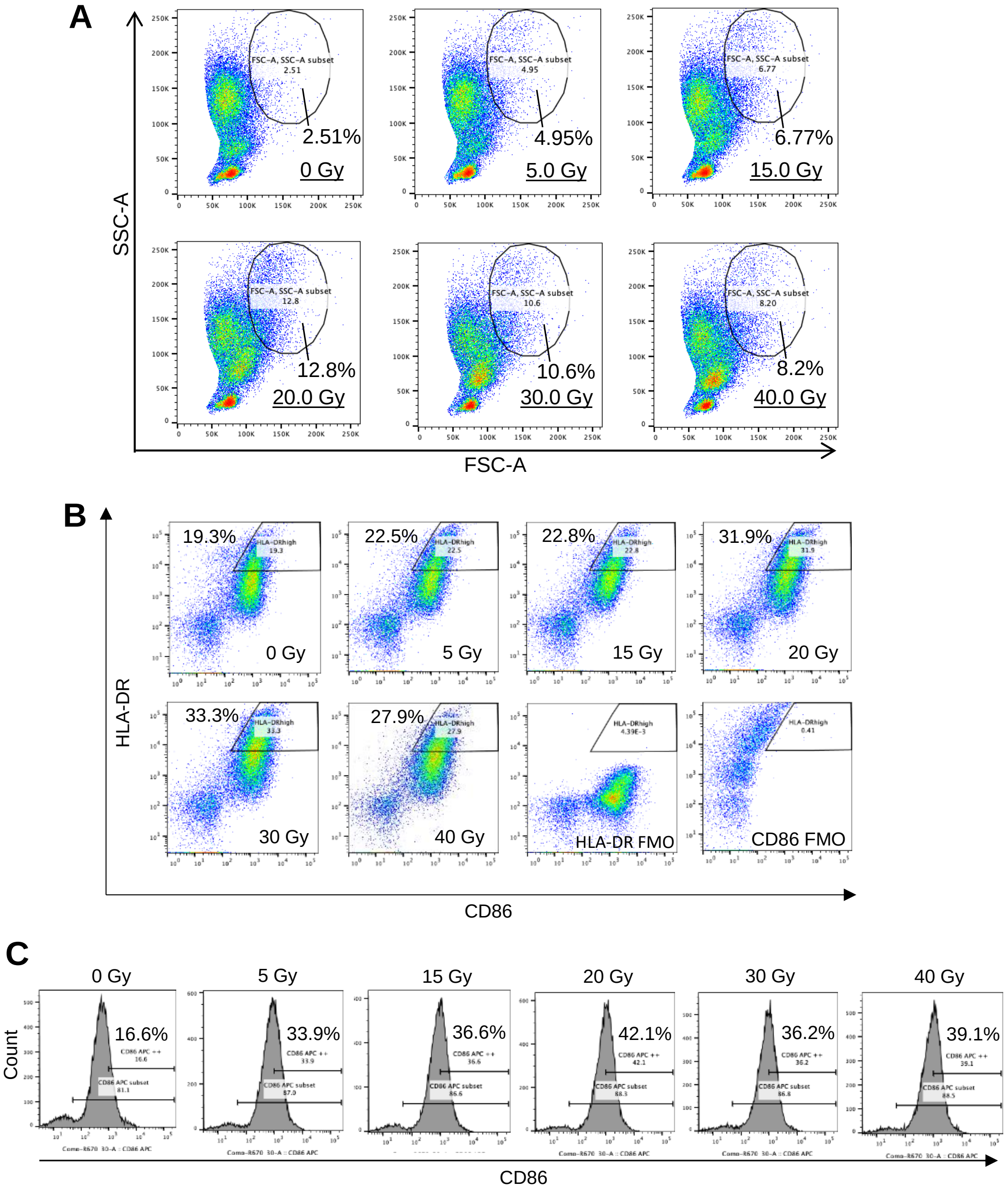

<u>Supplementary Figure 3</u>. Effect of gamma radiation on cyto-morphological parameters and presentation of activation markers on the surface of primary human monocytes.

(**A**) Analysis of the subset of large-size and high-granularity cells within the total population of elutriated primary human monocytes, exposed to indicated doses of γIR, 48 h post-exposure (Side scatter area: SSC-A – indirect measure of cell composition/granularity vs Forward scatter area FSC-A – indirect measurement of cells size); distribution of viable (DAPI-negative) cells. (**B**) Analysis of the subsets of “activated” (HLA-DR^{high}; CD86^{+}) myeloid cells in *ex vivo* irradiated cultures of human peripheral blood elutriated monocytes after exposure to indicated doses of gamma radiation, 48 h post-exposure. Gated on single, viable (DAPI-) cells. (**C**) Percent of CD86^{high} subset within the population of CD86^{+} myeloid cells in *ex vivo* irradiated cultures of human peripheral blood elutriated monocytes upon indicated doses of gamma radiation, 24 h post-exposure.

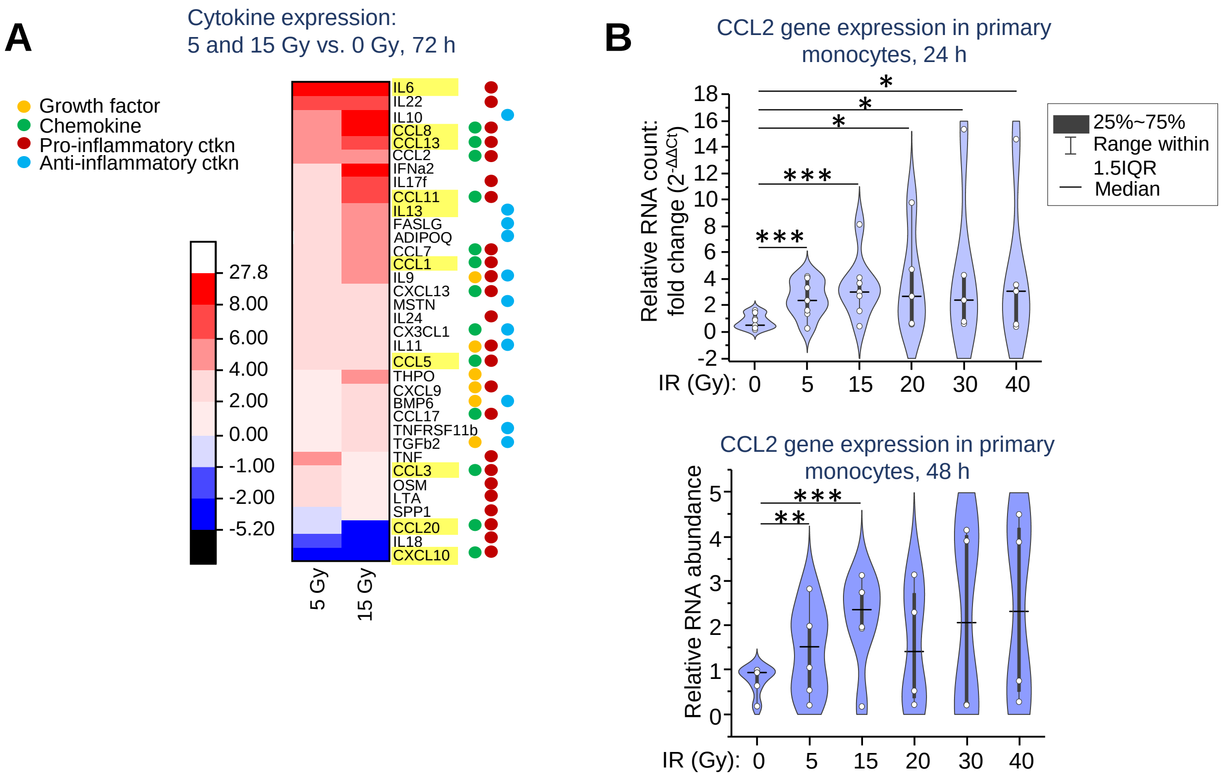

Supplementary Figure 4. Cytokine expression in THP1 monocytic cell line and primary human monocytes exposed to different doses of gamma-radiation.

(**A**) Heatmap depicting expression of differentially expressed cytokines (≥2-fold difference) in THP1 cells exposed to a 5 or 15 Gy γIR dose vs. non-irradiated cells, measured by RT^2 PCR array of total cellular RNA samples, 72h after γIR exposure: rows: cytokine gene codes; columns: samples. SASP marker genes are highlighted by yellow background. Color codes are shown on the separate left panel. The colored circles on the right mean: yellow – activity as a growth factor, green – chemokine, red – pro-inflammatory, blue – anti-inflammatory activities. (**B**) Expression of CCL2 gene in primary human monocytes exposed to indicated doses of γIR, measured by RT-qPCR vs. GAPDH and GUSB reference housekeeping genes at 24 h (upper panel) and 48 h (lower panel) post-exposure. Minimum number of biological samples is 5. * $p<0.05$, ** $p<0.01$, *** $p<0.001$, paired Wilcoxon test.

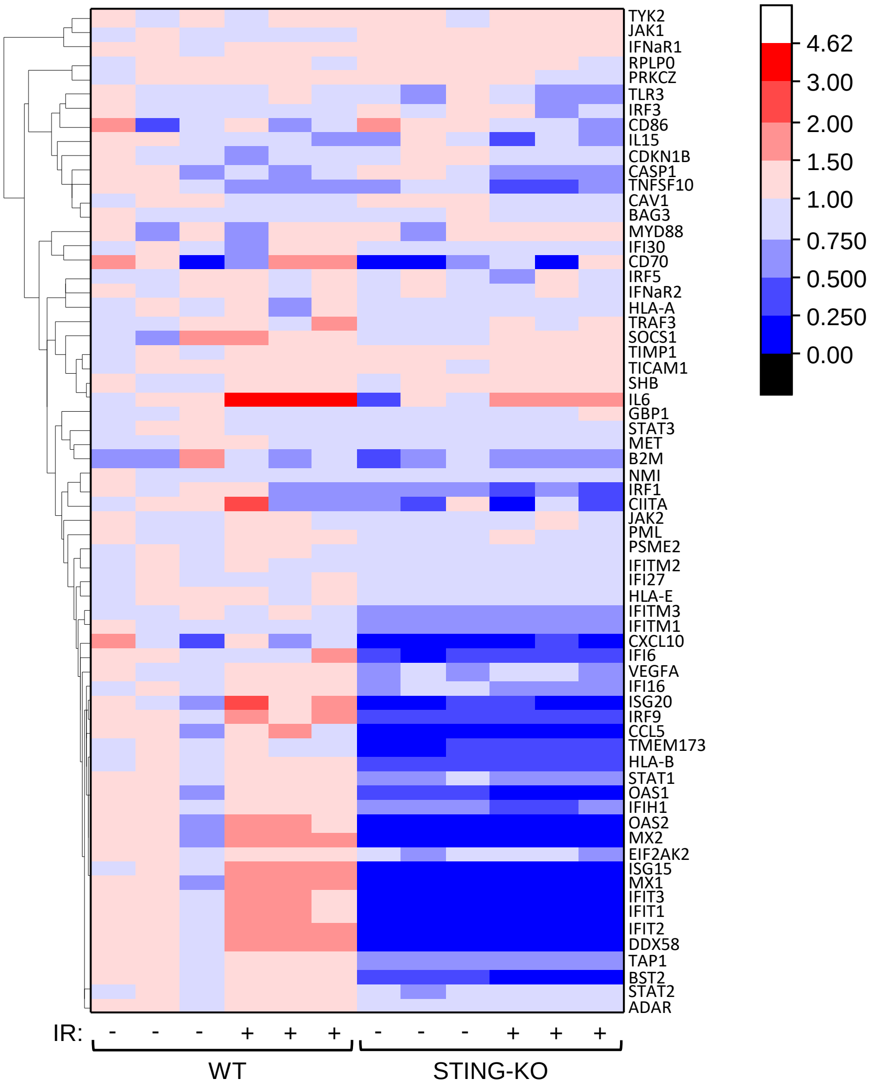

<u>Supplementary Figure 5</u>. Expression of multiple interferon-stimulated genes in irradiated FaDu head and neck squamous cell carcinoma cells is STING-dependent.

Heatmap depicting expression of interferon-stimulated genes (ISGs) in wild-type and STING-knockout FaDu cells exposed to 4 fractions of 2 Gy doses of X-ray, based on analysis of transcriptomic dataset GSE147085. Color codes are shown on the separate right panel.

2. Supplementary Tables

Excel supplementary tables:

<u>Supplementary Table 1</u>. Cytokine gene expression values ($_{\Delta\Delta}$Ct) in THP1 cells exposed to indicated radiation doses, measured by RT-qPCR analysis of triplicated samples using RT2 PCR array at 48- and 72-hours post-exposure.

<u>Supplementary Table 2</u>. Gene expression values ($_{\Delta\Delta}$Ct) of interferon-stimulated genes in THP1-Dual, THP1-Dual IFNAR-KO, THP1-Dual MAVS-KO, and THP1-Dual STING-KO cells measured by RT-qPCR analysis of triplicated samples using RT2 PCR array.

<u>Supplementary Table 3</u>. Gene expression: RNA-seq raw values in unexposed and irradiated (RT) FaDu head and neck squamous cell carcinoma cells with/without STING-knockout (STING-KO/WT).

<u>Supplementary Table 4</u>. CPM normalized values of gene expression in unexposed and irradiated (RT) FaDu head and neck squamous cell carcinoma cells with/without STING-knockout (STING-KO/WT).

<u>Supplementary Table 5</u>. Reagents and resources.

REAGENT or RESOURCE	SOURCE	IDENTIFIER
**Antibodies**		
β-Actin (clone AC-15)	Millipore Sigma	A5441
GAPDH (clone 0411)	Santa Cruz Biotechnology	sc-47724
CD11b (clone D12), APC	BD Biosciences	340936
CD206 (MMR), (clone 15-2), PE/Cy7	BioLegend	321124
CD86 (clone BU63), APC	BioLegend	374208
CD80 (B7-1), (clone 2D10.4), FITC, eBioscience	Thermo Fisher Scientific	11-0809-42
HLA-DR (clone LN3) – PE-Cyanine5, eBioscience	Thermo Fisher Scientific	15-9956-42
CD45RA (clone HI100)- PECy7	BD Pharmingen	560675
CD44 (clone 515)-PE	BD Pharmingen	550989
CD14 (clone M5E2)-APC	BioLegend	301808
CD16 (clone 3G8)- PE	BioLegend	302008
CD19 (clone HIB19)-FITC	BioLegend	302256

CD 66b (clone G10f5)-FITC	BioLegend	305104
CD20 (clone 2H7)-FITC	BioLegend	302304
CD3 (clone HIT3a)-FITC	BioLegend	300306
CD56 (NCAM) (clone MEM-188)-FITC	BioLegend	304604
IRF-3 (clone D6I4C)	Cell Signaling Technology	1190
NF-kB p65 (clone D14E12)	Cell Signaling Technology	8242
Phospho- NF-kB p65 (Ser536) (clone 93H1)	Cell Signaling Technology	3033
IFI-16 (clone 1G7)	Santa Cruz Biotechnology	Sc-8023
IFI16 (clone 2E3)	Abcam	ab55328
cGAS (rabbit polyclonal)	Abcam	ab224144
DDX41 (clone 2F4)	Novus Biological	H00051428-M01
MAVS (polyclonal)	Cell Signaling Technology	3993
Caspase-8 (clone 1C12)	Cell Signaling Technology	9746
MDA-5 (clone D74E4)	Cell Signaling Technology	5321
IFIH1/MDA-5 (rabbit polyclonal)	Proteintech	21775-1-AP
p21 $^{Waf1/Cip1}$ (clone 12D1)	Cell Signaling Technology	2947
Mek1/2 (clone D1A5)	Cell Signaling Technology	8727
LGP2 (clone D3I3L)	Cell Signaling Technology	12869
Histone H3 (clone D1H2)	Cell Signaling Technology	4499
Calnexin (clone C5C9)	Cell Signaling Technology	2679
RIG-1 (clone D33H10)	Cell Signaling Technology	4200

Sting (clone D2P2F)	Cell Signaling Technology	38866
Phospho-Sting (Ser366) (clone E9A9K)	Cell Signaling Technology	50907
Toll-like Receptor 3 (clone D10F10)	Cell Signaling Technology	6961
KAP1 (TRIM28)	Proteintech	66630-1-IG
Poly (ADP-Ribose) Polymerase (PARP)	Biosource International	AHF0262
Anti-DNA-RNA hybrid [S9.6]	Kerafast	ENH001
Pan-enterovirus 9D5 monoclonal	Millipore Sigma	3361
Anti-dsDNA [3519 DNA]	Abcam	Ab27156
Anti-mouse IgG, HRP-linked	Cell Signaling Technology	7076
Anti-rabbit IgG, HRP-linked	Cell Signaling Technology	7074
**Chemicals, commercial siRNA, Dyes, Peptides, and Recombinant Proteins**		
TRIzol Reagent	Thermo Fisher Scientific	15596026
Human BD Fc Block	BD Pharmingen	564220
RNase-free DNase Set	Qiagen	79256
RNeasy Mini kit	Qiagen	74106
RNase Recombinant Ribonuclease Inhibitor	Thermo Fisher Scientific	N8080119
Halt Protease Inhibitor Cocktail	Thermo Fisher Scientific	78429
Silencer Select Pre-Designed siRNA, Gene Symbol: DDX41	Thermo Fisher Scientific	s28122
Silencer Select Pre-Designed siRNA, Gene Symbol: cGAS	Thermo Fisher Scientific	s41748
Silencer Select Pre-Designed siRNA, Gene Symbol: IFI16	Thermo Fisher Scientific	s7136
Silencer Select Negative Control No. 1 siRNA	Thermo Fisher Scientific	4390843

Lipofectamine RNAiMAX reagent	Thermo Fisher Scientific	13778
GelCode Blue Safe protein Stain	Thermo Fisher Scientific	24594
Poly(dA:dT) / Lyovec - dsDNA naked – complexed with transfection reagent	InvivoGen	tlrl-patc
VACV-70/ LyoVec – dsDNA, CDS agonist complexed with transfection reagent	InvivoGen	tlrl-vav70c
LyoVec Cationic lipid for transfection of mammalian cells	InvivoGen	lyec-1
**Critical Commercial Assays**		
Quanti-Luc, Coelenterazine-based luminescence assay reagent	InvivoGen	rep-glc2
Quanti-Blue assay reagent	InvivoGen	rep-qbs2
RT^2 Profiler PCR Array Human Type I Interferon Response	Qiagen	330231 PAHS-016ZA
RT^2 Profiler PCR Array Human Cytokines and Chemokines	Qiagen	330231 PAHS-150ZA
MaqQuant Plus DNA V2, DNA normalization kit	MaqBio	MQP-50010
Pierce BCA Protein Assay Kit	Thermo Fisher Scientific	23225
Wizard Genomic DNA Purification kit	Promega	A1120
Subcellular Protein Fractionation kit for cultured cells	Thermo Fisher Scientific	78840
SsoAdvanced Universal SYBR Green Supermix	Bio-Rad	172-5272
Bio-Plex Pro Human Inflammation customized Screening Panel (10)	Bio-Rad	N/A
**Experimental Models: Cell Lines, primary cells**		
THP1 Human monocytes, acute monocytic leukemia	ATCC	ATCC-TIB-202
THP1-dual NF-κB-SEAP IRF_Luc reporter Monocytes	InvivoGen	thpd-nfis

THP1-dual-ko-IfnaR2 cells		InvivoGen	thpd-koifnar2
THP1-dual-ko-STING cells		InvivoGen	thpd-kostg
THP1-dual-ko-MAVS cells		InvivoGen	thpd-komavs
THP1-dual-ko-cGAS cells		InvivoGen	thpd-kocgas
THP1-dual-ko-IFI16 cells		InvivoGen	thpd-koifi16
Elutriated monocytes (fraction 5), *Homo sapiens*		NIH Blood Bank	
**Oligonucleotides**			
**Name**	**Sequence (5’→3’)**		
HERV-specific primers			
HERV-Kenv-F	CTGAGGCAATTGCAGGAGTT	(1)	
HERV-Kenv-R	GCTGTCTCTTCGGAGCTGTT		
Primers for cellular genes (human)			
hGAPDH-F	TGCACCACCAACTGCTTAGC	PrimerBank/Human (https://pga.mgh.harvard.edu/primerbank/)	
hGAPDH-R	GGCATGGACTGTGGTCATGAG		
B-actinF	GTGGGGCGCCCCAGGCACCA	PrimerBank/Human (https://pga.mgh.harvard.edu/primerbank/)	
B-actinR	CTCCTTAATGTCACGCACGATTTC		
GUSb-F	CACCAGGATCCACCTCTGAT	(2)	
GUSb-R	TCCAAATGAGCTCTCCAACC		
IFI16-F	GATGCCTCCATCAACACCAAGC	OriGene	HP208650
IFI16-R	CTGTTGCGTTCAGCACCATCAC	OriGene	HP208650
MDA5-F	GCTGAAGTAGGAGTCAAAGCCC	OriGene	HP214413
MDA5-R	CCACTGTGGTAGCGATAAGCAG	OriGene	HP214413
SOCS1-F	TTCGCCCTTAGCGTGAAGATGG	OriGene	HP207209
SOCS1-R	TAGTGCTCCAGCAGCTCGAAGA	OriGene	HP207209
STING-F	CCTGAGTCTCAGAACAACTGCC	OriGene	HP219304
STING-R	GGTCTTCAAGCTGCCCACAGTA	OriGene	HP219304

ISG15-F	CTCTGAGCATCCTGGTGAGGAA	OriGene	HP208303
ISG15-R	AAGGTCAGCCAGAACAGGTCGT	OriGene	HP208303
OAS2-F	GCTTCCGACAATCAACAGCCAAG	OriGene	HP229366
OAS2-R	CTTGACGATTTTGTGCCGCTCG	OriGene	HP229366
IFI27-F	CGTCCTCCATAGCAGCCAAGAT	OriGene	HP208651
IFI27-R	ACCCAATGGAGCCCAGGATGAA	OriGene	HP208651
VEGFa-F	TTGCCTTGCTGCTCTACCTCCA	OriGene	HP202779
VEGFa-R	GATGGCAGTAGCTGCGCTGATA	OriGene	HP202779
RIGI-F	CACCTCAGTTGCTGATGAAGGC	OriGene	HP210787
RIGI-R	GTCAGAAGGAAGCACTTGCTACC	OriGene	HP210787
TLR7-F	CTTTGGACCTCAGCCACAACCA	OriGene	HP212264
TLR7-R	CGCAACTGGAAGGCATCTTGTAG	OriGene	HP212264
VACV70-DNA	CCA TCA GAA AGA GGT TTA ATA TTT TTG TGA GAC CAT CGA AGA GAG AAA GAG ATA AAA CTT TTT TAC GAC T	IDT	N/A
VACV70-RNA_anti	rArGrU rCrGrU rArArA rArArA rGrUrU rUrUrA rUrCrU rCrUrU rUrCrU rCrUrC rUrUrC rGrArU rGrGrU rCrUrC rArCrA rArArA rArUrA rUrUrA rArArC rCrUrC rUrUrU rCrUrG rArUrG rG	IDT	N/A
**Software and Algorithms**			
SnapGene		www.snapgene.com	
GeneAssist™ Custom siRNA Builder		https://www.thermofisher.com/order/custom-genomic-products/tools/sirna/	
FlowJo		www.flowjo.com	
INSPIRE		www.amnis.com	

CFX Manager™ Software v3.1	https://www.bio-rad.com/en-us/product/previous-qpcr-software-releases?ID=OO2BB34VY&source_wt=cfx-manager-software_surl	
ImageJ	https://imagej.nih.gov/	
Bcl2fastq2 conversion software 2.20	https://support.illumina.com/downloads/bcl2fastq-conversion-software-v2-20.html	
FastQC	http://www.bioinformatics.babraham.ac.uk/projects/fastqc/	
Trimmomatic	http://www.usadellab.org/cms/?page=trimmomatic	
STAR	https://github.com/alexdobin/STAR	
Samtools	http://samtools.sourceforge.net/	
Ensembl	ftp://ftp.ensembl.org/pub/release-99/gtf/homo_sapiens/Homo_sapiens.GRCh38.99.gtf.gz	
TEtranscripts	http://labshare.cshl.edu/shares/mhammelllab/www-data/TEtranscripts/TE_GTF/GRCh38_rmsk_TE.gtf.gz	
TEcount	https://pypi.org/project/TEtranscripts/	
R	https://cran.r-project.org/	
OriginPro 2019	www.originlab.com	

ClustalW (CLCbio Genomics Workbench, v11)	www.qiagen.com	
JalView	https://www.jalview.org/	
**Other**		
RIPA buffer	Thermo Fisher Scientific	89900
Normocin	InvivoGen	Ant-nr-1
Nytran N45, Nylon Blotting Membrane	Whatman	10416194
RNAse H	New England Biolabs	M0297
Clarity Western ECL Substrate	Bio-Rad	1705080

References

1. Li W, Lee MH, Henderson L, Tyagi R, Bachani M, Steiner J, et al. Human endogenous retrovirus-K contributes to motor neuron disease. Science translational medicine. 2015;7(307):307ra153.
2. Zhang SY, Clark NE, Freije CA, Pauwels E, Taggart AJ, Okada S, et al. Inborn Errors of RNA Lariat Metabolism in Humans with Brainstem Viral Infection. Cell. 2018;172(5):952-65 e18.
